# Bioactive PLA Filament with Antibacterial and Ion-Releasing Properties for Additive Manufacturing of Bone Scaffolds: QbD-Guided Development

**DOI:** 10.3390/pharmaceutics18091055

**Published:** 2026-08-25

**Authors:** Anastassiya Khrustaleva, Azamat Yedrissov, Dmitriy Khrustalev, Ivan Chernykh, Aleksandr Samorodov, Saule Akhmetova, Artyom Savelyev, Marlen Kiikbayev, Polina Rusyaeva, Vladimir Kazantsev, Kristina Perepelitsyna, Sofiya Shapovalenko

**Affiliations:** 1 School of Pharmacy, Karaganda Medical University, 100008 Karaganda, Kazakhstan; anasteishin_2009@mail.ru (A.K.); savelevrtm@gmail.com (A.S.); rusyaevapolina@mail.ru (P.R.); wldmrrr@yandex.kz (V.K.); shapovalenko@qmu.kz (S.S.); 2 Department of Pharmacology, Ryazan State Medical University, 390026 Ryazan, Russia; ivchernykh88@mail.ru; 3 Translational Research Laboratory, All-Russian Scientific Research Institute of Medicinal and Aromatic Plants, 117216 Moscow, Russia; avsamorodov@gmail.com; 4 Department of Pharmacology, Sechenov First Moscow State Medical University, 119991 Moscow, Russia; 5 Department of Biomedicine, Karaganda Medical University, 100008 Karaganda, Kazakhstan; s.ahmetova@qmu.kz

**Keywords:** polylactic acid (PLA), additive manufacturing, fused deposition modeling (FDM), multifunctional composites, Quality by Design (QbD), biogenic calcium filler, mollusk shell, gentamicin, antibacterial activity, bioactive ion release, scaffold, bone regeneration

## Abstract

**Background/Objectives:** The development of multifunctional biomaterials for bone regeneration remains a key challenge in additive manufacturing. Although polylactic acid (PLA) is widely used in fused deposition modeling (FDM), its limited bioactivity and lack of intrinsic antibacterial functionality restrict its application in implantable constructs. This study aimed to develop a PLA-based composite filament combining ion-mediated bioactive potential and local antibacterial functionality using a Quality by Design (QbD) approach. **Methods:** PLA-based composite filaments incorporating a mollusk shell-derived biogenic calcium-containing filler (20 wt.%) and gentamicin (5 wt.%) were fabricated by solvent-free melt extrusion. A QbD framework was applied to define the Quality Target Product Profile (QTPP), identify critical quality attributes (CQAs), and assess critical material attributes (CMAs) and critical process parameters (CPPs). The material was characterized by SEM–EDS combined with ImageJ-based quantitative image analysis, TGA/DSC, mechanical testing, ICP-AES analysis of aqueous extracts, agar diffusion antibacterial assays, FDM printability assessment, and in vivo biocompatibility testing in a rat subcutaneous implantation model. **Results:** The developed PLA–Gen–MS material was obtained as a continuous filament with a diameter of 1.75 ± 0.05 mm and was successfully used for FDM printing of model scaffold structures. SEM–EDS confirmed matrix continuity and distribution of the calcium-containing mineral phase. ICP-AES revealed a calcium-dominant multicomponent ion release profile, with Ca as the predominant element and measurable levels of Sr, Mg, P, Mn, and Fe. TGA/DSC confirmed thermal compatibility of the components under melt-processing conditions. PLA–Gen–MS demonstrated antibacterial activity against all tested strains, with inhibition zones of approximately 20–21 mm. In vivo, the material showed a favorable preliminary tissue response compared with TiLOOP^®^, including faster reduction of inflammatory infiltration and absence of foreign body giant cells by day 14. **Conclusions:** The QbD-guided strategy enabled the development of a multifunctional PLA-based filament integrating melt processability, structural integrity, ion-mediated bioactive potential, antibacterial functionality, printability, and favorable preliminary biocompatibility. PLA–Gen–MS can be considered a promising platform for further development of personalized bioactive and antibacterial scaffold constructs for bone regeneration.

## 1. Introduction

Musculoskeletal diseases and traumatic injuries remain among the leading causes of disability, chronic pain, and reduced quality of life worldwide. According to the World Health Organization and the Global Burden of Disease Study, musculoskeletal disorders affect approximately 1.71 billion people and represent one of the primary contributors to global disability, accounting for approximately 149 million years lived with disability (YLDs), or about 17% of the global burden [1]. Increasing life expectancy, the rising prevalence of osteoporosis and degenerative bone conditions, including osteoarthritis, as well as the growing incidence of complex bone defects, are driving a steady increase in the number of patients requiring effective solutions for tissue reconstruction and replacement. According to projections based on the Global Burden of Disease (GBD) 2021 study, the global number of musculoskeletal disorder cases may reach 2.161 billion by 2035, while the category of other musculoskeletal disorders is expected to increase by approximately 115% by 2050 compared to 2020 [2,3]. Against this background, the development of next-generation materials for bone tissue engineering is critical to meet the growing clinical demand for personalized, functionally active, and safe implantable systems.

In recent decades, the concept of personalized medicine has gained increasing importance in this field, with additive manufacturing technologies playing a central role. Additive manufacturing enables the fabrication of patient-specific implants and tissue engineering scaffolds tailored to individual anatomical features [4]. Among the available technologies, fused deposition modeling (FDM) occupies a prominent position due to its technological simplicity, accessibility of equipment, scalability, and the ability to produce customized structures directly from thermoplastic filaments [5]. However, a key limitation of the field is that, despite the rapid progress in additive manufacturing technologies, the range of specialized bioactive and multifunctional filaments suitable for FDM printing remains extremely limited [5,6].

Polylactic acid (PLA) is one of the leading biodegradable polymers used in additive manufacturing for biomedical applications. PLA exhibits high biocompatibility, a predictable degradation rate, good processability, and has been approved for medical use by regulatory authorities, including the U.S. Food and Drug Administration [7]. Owing to these properties, PLA is widely regarded as a promising platform for the development of temporary scaffolds and implantable constructs in bone tissue engineering [8]. However, despite these advantages, this polymer also has well-recognized limitations, including low intrinsic bioactivity, limited ability to stimulate osteogenesis, and insufficient resistance to bacterial colonization, which significantly restrict its applicability in bone regeneration [9,10]. Therefore, PLA should not be considered a complete biomedical solution, but rather as a technologically convenient matrix requiring targeted functionalization.

The most common approaches to PLA modification involve the incorporation of calcium-containing compounds, such as hydroxyapatite, tricalcium phosphate, and bioactive glass, which impart osteoconductive properties to the material [11,12,13,14,15,16], as well as the introduction of antimicrobial agents—including antibiotics, metal nanoparticles (e.g., silver), or polymeric substances (e.g., chitosan)—aimed at suppressing pathogenic microflora and preventing biofilm formation [11,17]. However, most of these systems remain functionally limited and are primarily designed either to enhance bioactivity or to confer antibacterial properties, whereas clinically relevant implantable materials must simultaneously provide structural support, bioactive ionic stimulation, and localized antimicrobial protection [11,17].

Notably, a significant portion of research in this area is focused on the development of pastes, hydrogels, and suspensions for extrusion-based bioprinting, while studies specifically addressing thermoplastic filaments for FDM printing are considerably less common [18,19]. Moreover, multifunctional filaments capable of simultaneously providing bioactive ionic effects and localized antibacterial protection within a single material system remain relatively underexplored [11,20].

The choice of the mineral phase also has substantial scientific and practical significance. In most studies, synthetic calcium-containing fillers—primarily hydroxyapatite, tricalcium phosphate, and bioactive glass—are employed [11,21,22]. These materials are widely used because they mimic, to different degrees, the calcium phosphate nature of the mineral phase of bone and provide osteoconductive support. However, the present work explores a different strategy based on a shell-derived biogenic calcium carbonate phase rather than on the direct incorporation of preformed calcium phosphate fillers. In contrast, biogenic calcium-containing materials, particularly mollusk shells, can be regarded as complex mineral–organic systems capable not only of supplying Ca^2+^ ions but also of providing additional trace elements, including Mg^2+^ and Sr^2+^, which may influence cellular responses and regenerative processes [23,24]. In this regard, mollusk shells are of interest as accessible CaCO_3_-based biogenic mineral fillers that may serve a dual role as both structural components and sources of a calcium-dominant multicomponent ionic profile. This distinguishes the proposed mineral phase from conventional synthetic calcium-phosphate fillers and provides an additional functional dimension to the composite beyond simple mineral reinforcement. However, their chemical and structural complexity, as well as variability in composition and properties, necessitate a systematic approach to the design of such composites, taking into account the interrelationships between raw material characteristics, processing parameters, and target material properties.

Under these conditions, the application of the Quality by Design (QbD) approach becomes particularly relevant as a framework for the rational design of multifunctional composite systems [25,26]. In contrast to traditional empirical selection of composition and processing conditions, QbD focuses on the predefinition of the target product profile (QTPP), the identification of critical quality attributes (CQA), and the analysis of critical material attributes (CMA) and critical process parameters (CPP) [25,26]. This rationale aligns with the broader engineering paradigm, where the reproducibility of the final product depends not only on the selection of raw materials but also on rigorous control of process parameters [27,28]. For composites containing biogenic fillers, QbD is especially important as a risk-based framework that enables the integration of raw material variability, processing conditions, and target properties into a unified development strategy [25,26].

The scientific novelty of the present study arises from the integration of three complementary features within a single PLA-based filament platform. First, the mineral component is represented by a shell-derived biogenic CaCO_3_-based phase rather than by conventional synthetic calcium-phosphate or bioactive-glass fillers. In this system, the biogenic mineral phase functions not only as a structural filler but also as a source of a calcium-dominant multicomponent ionic profile. Second, this mineral phase is combined with gentamicin within the same PLA matrix, thereby integrating ion-releasing and antibacterial functionalities in a single melt-processable filament while preserving FDM printability. Third, the composition and processing conditions were developed within a QbD framework linking the QTPP, CQAs, CMAs, and CPPs with subsequent experimental verification of the resulting material. This approach enables the PLA–Gen–MS system to be evaluated within an integrated “composition–processing–structure–function” paradigm encompassing compositional reproducibility, microstructural organization, thermal and mechanical behavior, ion release, antibacterial activity, printability, and preliminary in vivo biocompatibility. The aim of this study was to develop and experimentally validate this multifunctional filament for FDM fabrication of bioactive and antibacterial bone scaffolds.

## 2. Materials and Methods

### 2.1. QbD Concept and Risk Assessment

The development of the multifunctional PLA-based filament was carried out using the Quality by Design (QbD) approach in accordance with the ICH Q8 guideline [29]. Within this framework, the material was considered not as a broad exploratory composite system, but as a targeted filament formulation intended for FDM fabrication of biodegradable implantable constructs with antibacterial functionality and ion-releasing properties.

The Quality Target Product Profile (QTPP) was defined taking into account the intended biomedical application of the material, the requirements for filaments used in additive manufacturing, and the key functional properties expected from the selected composition (Table 1) [29,30].

Based on the QTPP, the critical quality attributes (CQAs) of the target filament were identified. These included filament geometry, structural homogeneity, thermal properties, thermal stability, mechanical integrity, ion release profile, antibacterial activity, and biocompatibility (Table 2).

Identification of the CQAs enabled the establishment of a direct relationship between the intended product profile and the measurable properties of the developed filament, thereby providing a foundation for rational material design and subsequent experimental validation. This framework also supported a systematic understanding of the factors governing final product quality and facilitated a risk-based development strategy.

Factors potentially influencing the quality of the target filament were classified as critical material attributes (CMAs) and critical process parameters (CPPs). Particular attention was given to the polymer molecular weight, mineral filler particle size, mineral phase content, gentamicin loading, extrusion temperature, extrusion rate, and material residence time in the heating zone (Table 3).

A qualitative risk assessment was then performed to determine the relative influence of each CMA and CPP on the selected CQAs in accordance with the principles of QbD and quality risk management [29,30,31]. The individual risk assignments were based on established knowledge of PLA processing and the physicochemical properties of the composite components, published experimental evidence, and preliminary formulation and extrusion observations obtained during development of the PLA–Gen–MS system. Risk levels were categorized as Low, Medium, Medium–High, or High according to the expected magnitude of the influence of each factor on the corresponding CQA. In particular, the assessment considered the reported influence of PLA molecular weight on melt rheology, crystallization, and mechanical behavior [32,33], mineral filler particle size and content on the morphology and properties of PLA-based composites [34], screw speed and filler content on filler dispersion during PLA melt compounding [35], processing temperature and residence time on melt-phase degradation of PLA [36], and gentamicin incorporation and thermal processing on the antibacterial functionality of PLA-based filaments and constructs [37].

**Figure 1:**
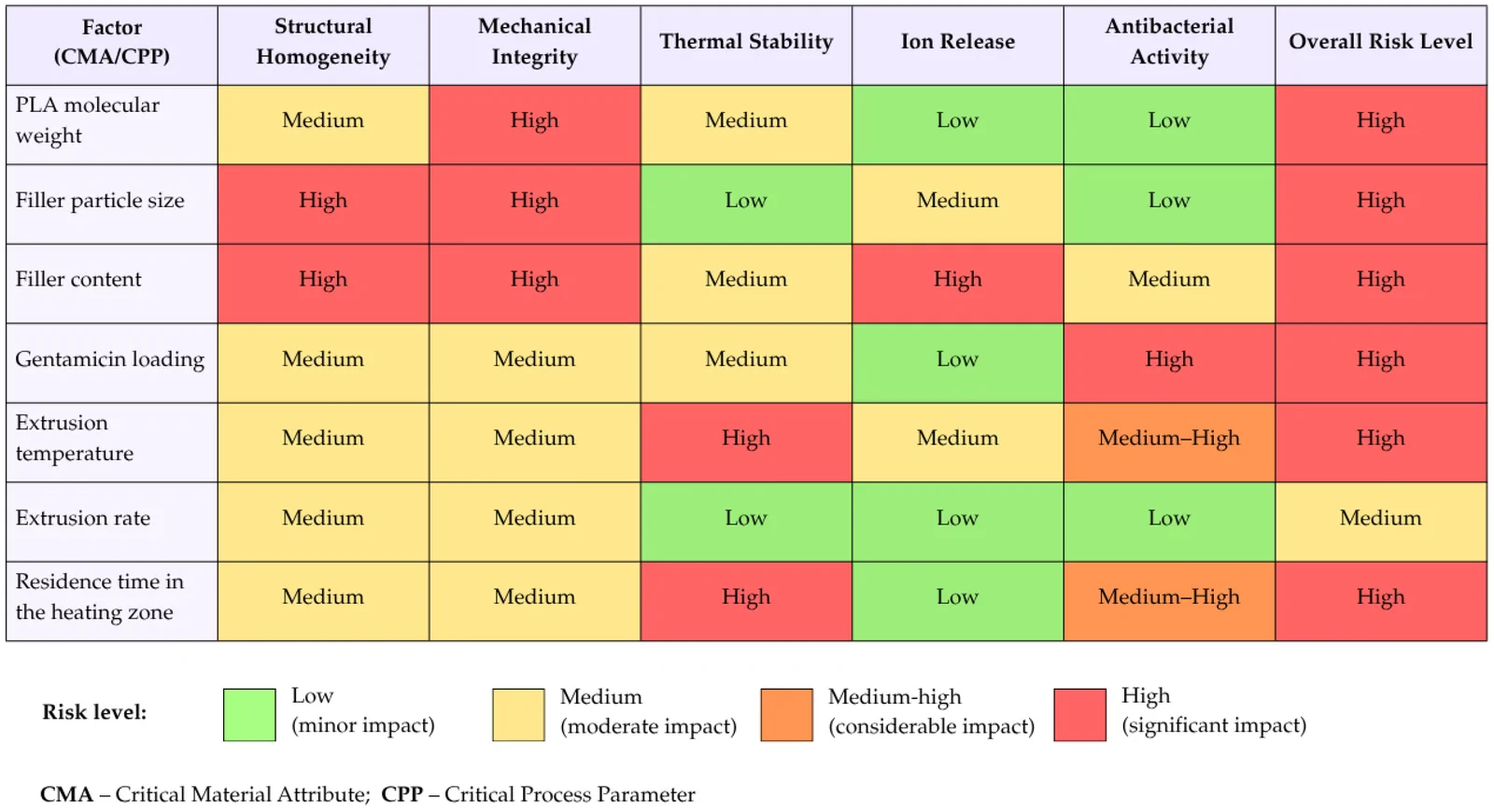
Qualitative risk assessment heatmap illustrating the influence of CMAs and CPPs on the selected CQAs. The Overall Risk Level corresponds to the highest individual risk level assigned to each CMA or CPP across the evaluated CQAs.

The Overall Risk Level for each CMA or CPP was determined using a conservative maximum-risk approach, whereby the highest individual risk level assigned to that factor across the evaluated CQAs was taken as its overall risk level. This approach was used to ensure that a potentially critical influence on an individual CQA was not obscured by lower risk ratings for other quality attributes. The results are presented in Figure 1.

The risk assessment showed that PLA molecular weight, mineral filler particle size and content, gentamicin loading, extrusion temperature, and material residence time in the heating zone were the most significant factors determining the quality of the target filament. Parameters associated with thermal exposure are of particular importance, as they may affect both the stability of the PLA matrix and the preservation of the antibacterial functionality of gentamicin.

These findings were subsequently used, together with literature evidence and preliminary formulation and extrusion studies, to define the target composition for experimental validation. Published studies have demonstrated the feasibility of incorporating calcium-based mineral fillers into PLA-based systems over a relatively broad concentration range, including biogenic CaCO_3_-containing formulations with mineral loadings up to 20 wt.% and other calcium-based fillers at comparable or higher concentrations [38,39,40]. In the preliminary formulation studies performed during development of the present system, different mineral filler contents were evaluated, and 20 wt.% was identified as the most suitable loading for providing a substantial CaCO_3_-based mineral fraction while preserving a continuous PLA matrix, stable filament formation, and melt processability. Gentamicin was incorporated at 5 wt.% based on literature data on local antibiotic-loaded orthopedic biomaterials and biodegradable polymer-based systems [41,42], together with preliminary formulation observations. The remaining 75 wt.% PLA provided the continuous thermoplastic matrix required for melt extrusion and subsequent FDM processing. Accordingly, the 75:20:5 PLA:MS:Gen ratio was selected as a balanced target formulation combining processability, bioactive mineral loading, and local antibacterial functionality.

### 2.2. Materials

Polylactic acid (PLA, Ingeo 4032D, NatureWorks LLC, Plymouth, MN, USA) in pellet form was used as the polymer matrix. Ingeo 4032D was selected because this specific PLA grade has previously been used in experimental biomedical studies involving PLA-based scaffolds and bone-related implant materials [43,44,45]. Its well-characterized melt-processing behavior and documented use in experimental scaffold development also supported its selection for the present proof-of-concept study. Crushed mollusk shells were used as the mineral filler, representing a natural calcium-containing biogenic material. The shell material was commercially purchased from ASKOR AgroTrade LLC (Buynaksk, Russia). The shells were pre-cleaned, washed with distilled water, and dried at 100 °C, followed by mechanical grinding to obtain a powder.

Gentamicin sulfate (Sigma-Aldrich, St. Louis, MO, USA) was used as the antibacterial agent. The target PLA–Gen–MS composition used in the main experiments contained 75 wt.% PLA, 20 wt.% mollusk shell-derived mineral filler, and 5 wt.% gentamicin. A control composite without gentamicin, containing PLA and the mineral filler, was designated as PLA–MS. The number, geometry, and preparation of specimens used for individual tests are specified in the corresponding methodological sections.

Standard microbial test strains were used for microbiological studies: *Staphylococcus aureus* ATCC 6538, *Escherichia coli* ATCC 25922, and *Pseudomonas aeruginosa* ATCC 27853. Deionized water was used for the preparation of composite extracts for elemental analysis.

### 2.3. Fabrication of the Bioactive Composite Filament

The bioactive PLA–Gen–MS composite filament was produced by solvent-free melt extrusion.

Before preparation of the composite blend, the initial PLA pellets were subjected to preliminary mechanical processing to obtain a polymer fraction suitable for dry blending with the powdered mineral filler derived from mollusk shells and gentamicin sulfate. For this purpose, the PLA pellets were first formed into thin plates using a laboratory hot press at 180 °C. After cooling to room temperature, the plates were manually cut into small fragments. This intermediate step facilitated subsequent knife milling, since thin plate-like fragments were more readily captured by the cutting elements of the mill than the original pellets. Although the hot-pressing step used here was introduced as a practical pre-processing step to facilitate milling, preliminary size reduction of PLA by knife or Wiley milling has previously been reported in PLA-based blend and composite processing [46].

The obtained fragments were milled using a laboratory knife mill GRINDOMIX GM 200 (Retsch GmbH, Haan, Germany). The process was carried out intermittently to prevent overheating of the polymer. The material was then sieved through a 500 µm mesh, and only the fraction passing through the sieve was used for composite preparation. Representative photographs of the main stages of PLA-fraction preparation are presented in the Supplementary Materials (Figure S1). The morphology of the sieved PLA fraction was additionally evaluated by optical microscopy.

The prepared PLA fraction was combined with mollusk shell powder and gentamicin sulfate at a weight ratio of 75:20:5 and dry-mixed manually in a laboratory mortar for approximately 5 min. During mixing, the material was periodically redistributed from the walls and bottom of the mortar to promote uniform distribution of the powdered components throughout the PLA fraction. The resulting dry blend was visually inspected for macroscopic uniformity and subsequently subjected to quantitative compositional homogeneity assessment as described in Section 2.4.

The obtained dry composite blend was processed using a laboratory desktop single-screw filament extruder (Wellzoom desktop filament extruder B, Wellzoom, Shenzhen, China) equipped with two independently controlled heating zones. Under the selected processing conditions, the extrusion temperature was 172 °C and the screw rotation speed was 2 rpm. The dry blend was gradually fed into the extruder to maintain stable material feeding during extrusion. These parameters were selected based on preliminary technological trials as conditions enabling the production of a continuous filament while limiting excessive thermal exposure of the PLA matrix and gentamicin.

A continuous filament was formed at the extruder outlet, cooled to room temperature, and used for subsequent physicochemical, microstructural, functional, and biological studies. The resulting filament had a nominal diameter of 1.75 mm and remained within the acceptable range required for subsequent FDM printing. The filament diameter was controlled at several points along the length of the extruded material.

### 2.4. Assessment of the Compositional Homogeneity of the Dry Blend and the Resulting Filament

The compositional homogeneity of the PLA–Gen–MS system was evaluated at two technological stages: after dry blending, before loading the material into the extruder, and after production of the extruded PLA–Gen–MS filament. This approach made it possible to separately characterize the homogeneity of the initial dry blend and the compositional reproducibility of the final filament along its length.

For analysis of the dry blend, seven samples were collected from different regions of the blend volume after mechanical mixing. For analysis of the resulting filament, seven segments were collected from different regions of the extruded filament. Before extraction, the filament segments were cut into small fragments to increase the accessibility of gentamicin sulfate to the extraction medium. All samples were analyzed independently. In both cases, the distribution of the mineral component and gentamicin sulfate was evaluated.

The distribution of the mineral component was assessed by thermogravimetric analysis based on the residual mass at 600 °C. The analysis was performed under the conditions described in the thermogravimetric analysis section. For the purpose of homogeneity assessment, the residual mass at 600 °C was used as a compositional indicator of the thermally stable residue, which was predominantly associated with the mineral filler derived from mollusk shells.

The gentamicin sulfate content was determined spectrophotometrically after extraction of the antibiotic from dry-blend samples or filament segments. Extraction was performed with distilled water for five days in a thermostat at 37 °C with periodic mixing. After extraction, insoluble components were removed by filtration.

Quantitative determination of gentamicin was performed using a reaction with ascorbic acid in DMSO according to a previously published method [47]. Absorbance was measured at 365 and 510 nm. The gentamicin concentration was calculated from the calibration curve and expressed as wt.% relative to the initial sample mass.

To evaluate compositional homogeneity, the value range, mean, standard deviation (SD), and relative standard deviation (RSD, %) were calculated separately for the residual mass at 600 °C and for the gentamicin sulfate content. RSD was calculated using the following equation:

RSD = SD/Mean × 100%

### 2.5. Scanning Electron Microscopy, SEM–EDS, and Quantitative Image Analysis

The microstructure of the PLA–Gen–MS filament was examined by scanning electron microscopy (SEM) using a Helios 5 CX SEM/FIB system (Thermo Fisher Scientific, Waltham, MA, USA). The analysis was performed on cross-sections of the extruded filament prepared perpendicular to its longitudinal axis. To evaluate the overall morphology, cross-sectional geometry, and distribution of the mineral phase, images were acquired at different magnifications.

Morphological analysis was performed in backscattered electron contrast (BEC) mode, since calcium-containing mineral inclusions appeared as brighter regions against the PLA matrix in this imaging mode. Elemental analysis and mapping were performed using the integrated energy-dispersive X-ray spectroscopy (EDS) system of the Helios 5 CX SEM/FIB (Thermo Fisher Scientific, Waltham, MA, USA). Elemental distribution maps of the main elements (C, O, and Ca) were obtained from regions corresponding to the SEM images under standard detection conditions.

Quantitative analysis of the mineral-phase distribution was performed using the ImageJ software package (ImageJ 1.54p; National Institutes of Health, Bethesda, MD, USA). BEC-SEM images of the filament cross-section were used for analysis, as the mineral phase showed enhanced contrast relative to the polymer matrix. The images were calibrated using the scale bar, converted to 8-bit format, and processed using a fixed thresholding protocol. The mineral phase was segmented as the bright phase in the BEC images, followed by generation of a binary mask. The Watershed algorithm was applied to separate touching or closely adjacent objects.

For the entire cross-section, the number of detected mineral objects, total mineral-phase area, mineral-phase area fraction (%Area), and median object size expressed as D50 were calculated. In addition, a sensitivity analysis with respect to the minimum object area included in the analysis was performed to evaluate the contribution of the fine fraction to the total number of detected particles and the total mineral-phase area.

The spatial distribution of the filler was additionally evaluated using 16 equal-area circular regions of interest (ROIs) placed in different regions of the filament cross-section. For each ROI, the mineral-phase area fraction, number of objects, and size parameters D10 and D50 were determined. This approach was used to assess the local variability of filler distribution and to identify possible regions depleted in the mineral phase.

### 2.6. Thermogravimetric Analysis (TGA)

The thermal stability and degradation behavior of neat polylactic acid (PLA), mollusk shell powder (MS), gentamicin, and the PLA–Gen–MS composite were investigated by thermogravimetric analysis (TGA) using an STA 6000 simultaneous thermal analyzer (PerkinElmer, Shelton, CT, USA).

Measurements were conducted over a temperature range of 30–600 °C at a heating rate of 10 °C/min under an argon atmosphere. The sample mass was 20–30 mg.

TG/DTG data, the onset temperature of the main thermal degradation stage (T_onset_), the temperature corresponding to 5% mass loss (T_5_%), the temperature of maximum decomposition rate (T_max_), and the residual mass at 600 °C were determined. The residual mass was used for the semi-quantitative estimation of the contribution of the thermally stable mineral phase to the composite composition.

### 2.7. Differential Scanning Calorimetry (DSC)

The thermal transitions of neat polylactic acid (PLA), mollusk shell powder (MS), gentamicin, and the composite filament PLA–Gen–MS were evaluated using the heat-flow/DTA signal recorded during simultaneous thermal analysis on the STA 6000 thermal analyzer. The analysis was performed over a temperature range of 30–600 °C at a heating rate of 10 °C/min under an argon atmosphere. From the recorded thermal signal, the glass transition temperature (Tg), cold crystallization temperature (Tc), and melting temperature (Tm) were determined for PLA and the composite filament.

### 2.8. Mechanical Testing

The mechanical properties of neat PLA and the target PLA–Gen–MS composite were evaluated using three-point bending and tensile testing.

Three-point bending tests were conducted on a universal testing machine (MTS Criterion Model 43, MTS Systems Corporation, Eden Prairie, MN, USA) in accordance with ASTM D7264 [48]. Rectangular specimens with dimensions of 100 × 10 × 1.0 mm were fabricated by FDM printing from the corresponding neat PLA and PLA–Gen–MS filaments. Testing was performed at 23 °C and 50% relative humidity. The crosshead speed was 1 mm/min, and the load was applied at the midpoint between the supports. Flexural strength ($\sigma_{f}$) was calculated from the maximum load recorded during the test according to:

$$\sigma_{f}=\frac{3F_{max}\;L}{2bh^{2}}$$

where *Fmax* is the maximum load (N), *L* is the support span (mm), *b* is the specimen width (mm), and *h* is the specimen thickness (mm).

Tensile tests were performed using a universal testing machine (Tinius Olsen H25S, Tinius Olsen, Horsham, PA, USA) in accordance with ASTM D638 [49], with modifications for filament-type specimens. Segments of extruded neat PLA and PLA–Gen–MS filaments, with a nominal diameter of 1.75 mm, were used as test specimens. The gauge length was set to 20 mm. Tests were conducted at 23 °C and 50% relative humidity, with a crosshead speed of 2 mm/min. Tensile strength ($\sigma_{t}$) was calculated as the maximum tensile load divided by the initial cross-sectional area of the filament:

$$\sigma_{t}=\frac{F_{max}}{A_{0}}$$

where *Fmax* is the maximum tensile load (N) and *A*_0_ is the initial cross-sectional area of the filament (mm^2^).

At least five specimens of each material (neat PLA and PLA–Gen–MS) were tested for each type of mechanical test. The results were reported as mean ± standard deviation (SD).

### 2.9. Elemental Composition of the Aqueous Extract

The elemental composition of the aqueous extract of the PLA–Gen–MS composite was evaluated by inductively coupled plasma atomic emission spectroscopy (ICP-AES) within the framework of chemical characterization of potentially released inorganic constituents, consistent with the principles of ISO 10993-18 [50]. For extract preparation, the extruded PLA–Gen–MS filament was cut into approximately 10 mm-long segments. The extract was prepared using deionized water at a material-to-extraction-medium ratio of 1 g per 10 mL. Samples were incubated at 37 °C for 72 h, with the extraction temperature and duration selected with reference to the extraction conditions described in ISO 10993-12 [51].

After incubation, the aqueous extract was separated from the samples and subjected to quantitative elemental analysis by ICP-AES. A total of 41 elements were analyzed, including Ca, Sr, P, Mg, Mn, Fe, and other trace elements. The concentrations of the detected elements were expressed in mg/dm^3^.

### 2.10. Antibacterial Activity Assessment

The antibacterial activity of the composite materials was evaluated using the agar diffusion method with standard bacterial test strains: *Staphylococcus aureus* ATCC 6538, *Escherichia coli* ATCC 25922, and *Pseudomonas aeruginosa* ATCC 27853.

Bacterial suspensions were prepared by adjusting the cell concentration to a 0.5 McFarland standard (1.5 × 10^8^ CFU/mL) in accordance with EUCAST recommendations for the disk diffusion method [52]. The resulting suspensions were uniformly spread over the surface of agar plates using the lawn culture method.

Composite specimens (PLA–MS and PLA–Gen–MS) were prepared as discs 6 mm in diameter and 1 mm in thickness and placed on the surface of the inoculated agar. Standard gentamicin sulfate disks (10 µg/disk; BIOANALYSE Limited, Ankara, Turkey) were used as a positive control, while PLA–MS composite samples without antibiotic served as a negative control.

Incubation was carried out at 37 °C for 24 h. After incubation, the diameters of bacterial growth inhibition zones around the samples were measured using a calibrated digital caliper (Stayer, Pinto, Madrid, Spain; resolution 0.01 mm).

All measurements were performed in at least three independent replicates, and the results were reported as mean ± standard deviation (SD).

### 2.11. In Vivo Biocompatibility Assessment

The biocompatibility of the developed PLA–Gen–MS composite material was evaluated using a subcutaneous implantation model in laboratory rats. PLA–Gen–MS specimens for implantation were fabricated by FDM printing as rectangular plates with dimensions of 10 × 10 × 3 mm. Prior to implantation, the PLA–Gen–MS specimens were subjected to ultraviolet (UV) irradiation for 30 min. The study included 30 outbred white rats weighing 200–250 g, with equal sex distribution. All procedures were performed in accordance with ISO 10993-6 [53] principles and were approved by the Local Bioethics Committee of Karaganda Medical University, Protocol No. 2 dated 22 January 2025.

The animals were randomly assigned to three experimental groups: (i) sham operation (negative control); (ii) implantation of a commercially available titanium-coated polypropylene mesh, TiLOOP^®^ (pfm medical titanium GmbH, Nuremberg, Germany) (reference control); and (iii) implantation of the developed PLA–Gen–MS composite material.

Surgical procedures were performed under general anesthesia using xylazine (20 mg/kg, intramuscularly) and propofol (10 mg/kg, intravenously). After preparation of the surgical field, a longitudinal incision was made along the dorsal midline, a subcutaneous pocket was created by blunt dissection, and the corresponding implant material was placed into the pocket. The wound was closed using 3-0 absorbable polyglycolic acid suture material and covered with a sterile dressing.

During the postoperative period, the animals were housed individually with free access to food and water. No intraoperative or early postoperative complications were observed.

Animals were euthanized at predefined time points: days 1, 3, 7, 10, and 14 after implantation. At each time point, two animals from each experimental group were analyzed. The implantation area was excised en bloc, and the presence of adhesions was recorded macroscopically. Tissue samples were fixed in 10% neutral buffered formalin, processed according to standard histological procedures, embedded in paraffin, sectioned at a thickness of 4 μm, and stained with hematoxylin and eosin (H&E).

Histological evaluation was performed using digital image analysis. Morphometric assessment included measurement of the thickness of inflammatory infiltration and the area of tissue lysis. In addition, neovascularization, fibrosis, and the severity of the inflammatory response were evaluated using a semi-quantitative scale from 0 to 3, where a higher score corresponded to greater severity of the respective feature; the scoring criteria are provided in Supplementary Table S1.

### 2.12. FDM Printability Assessment

The FDM printability of the PLA–Gen–MS filament was evaluated using a Zonestar Z6 3D printer (Zonestar, Shenzhen, China) equipped with a 0.4 mm nozzle. The extruded PLA–Gen–MS filament with a nominal diameter of 1.75 mm was used directly for printing.

Printability was assessed by fabricating digital models with different levels of geometric complexity, including regular lattice scaffolds and a representative anatomical model. The digital models were prepared for printing and converted into printer-compatible toolpaths using PrusaSlicer software (version 2.8.1; Prusa Research, Prague, Czech Republic).

The printing parameters were selected on the basis of preliminary printing trials and the thermal and processing characteristics of the PLA–Gen–MS filament. A nozzle temperature of 190 °C was selected to provide stable melt flow and continuous material extrusion while limiting excessive thermal exposure of the composite. The build-plate temperature was set to 50 °C to promote adhesion of the initial layers. A printing speed of 40 mm/s was selected as a compromise between stable filament deposition and preservation of the intended geometry. A 0.4 mm nozzle and a layer height of 0.2 mm were used to provide continuous material deposition and reproducible formation of the model structures. The main FDM processing parameters are summarized in Table 4.

### 2.13. Statistical Analysis

Statistical analysis was performed using Statistica 10.0 software (StatSoft Inc., Tulsa, OK, USA). Quantitative data were first assessed for normality using the Shapiro–Wilk test. Mechanical and antibacterial activity data are presented as mean ± standard deviation (SD). For morphometric histological data, normality was not confirmed; therefore, these data are presented as median and interquartile range, Me (Q1–Q3). Intergroup comparisons of morphometric parameters were performed using the Mann–Whitney U test. Differences were considered statistically significant at *p* < 0.05.

## 3. Results

Within the risk-based QbD approach described in Section 2.1, the target PLA–Gen–MS composition containing 20 wt.% biogenic mineral filler derived from mollusk shells and 5 wt.% gentamicin was selected for comprehensive experimental evaluation. This formulation was considered a multifunctional system combining the potential for ion-releasing properties, local antibacterial protection, and technological suitability for producing a continuous filament for FDM printing. The structural, thermal, mechanical, functional, and biological characteristics of the developed material are presented below.

### 3.1. Size Control of the PLA Fraction and Homogeneity of the Dry Blend Prior to Melt Extrusion

**Figure 2:**
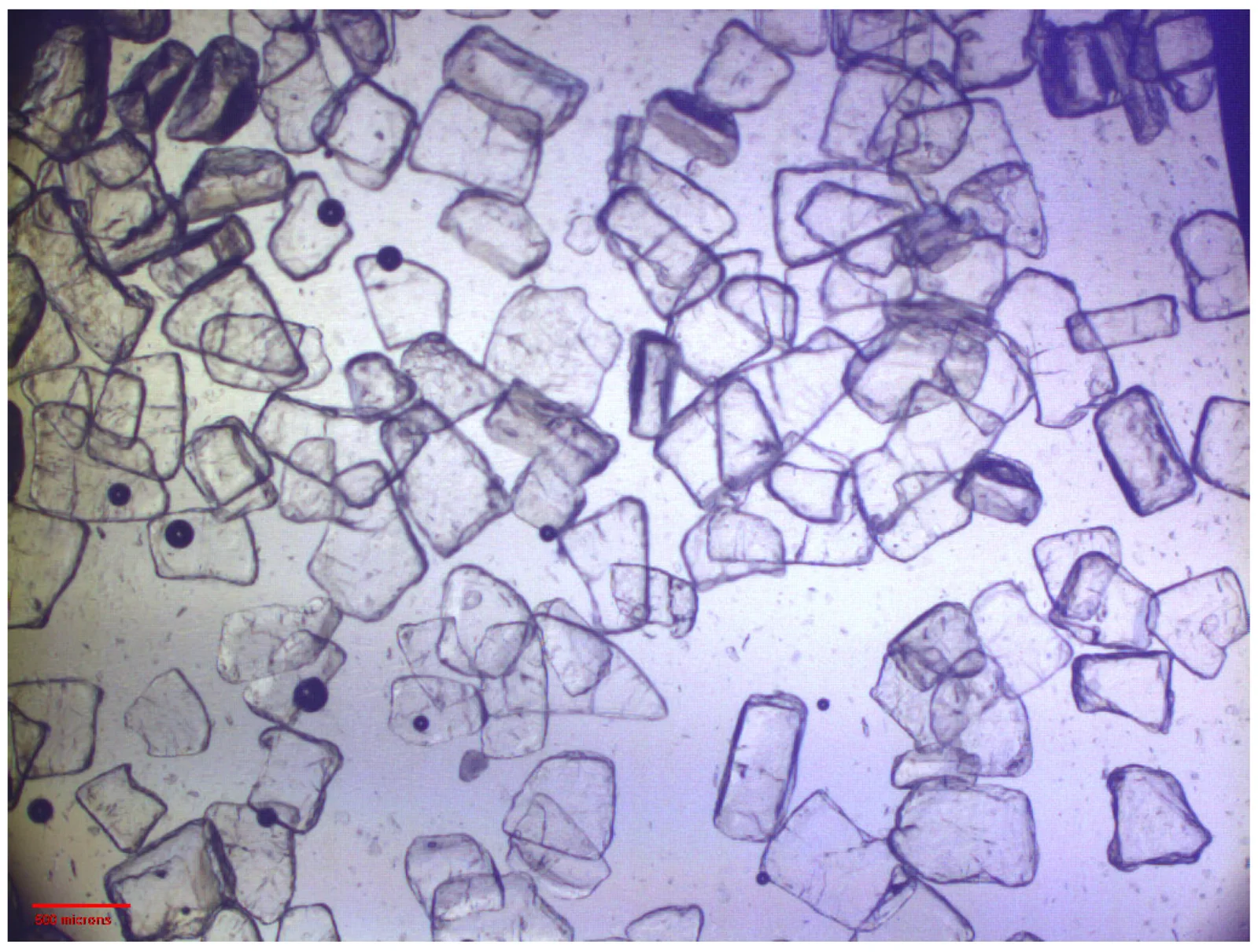
Optical microscopy of size-reduced PLA particulates.

Optical microscopy was performed to visually characterize PLA after mechanical size reduction and sieving. The sieved PLA fraction consisted of transparent fragmented plate-like particles, which were characteristic of the fracture of pre-formed PLA plates during knife milling (Figure 2). The original PLA pellets were not observed in the micrographs. These data confirm that, before preparation of the dry blend, the polymer component was converted from pelletized raw material into a dispersed size-controlled fraction, with particle passage additionally limited by a 500 μm mesh sieve.

Representative optical micrograph of PLA particulates after knife milling and sieving through a 500 μm sieve. The image shows fragmented plate-like PLA particles obtained from the pre-pressed polymer plates. Scale bar: 500 μm.

The homogeneity of the dry PLA–Gen–MS blend was additionally evaluated before the melt extrusion stage. This step was included in the study because the components of the composition differ substantially in physicochemical nature, density, morphology, and particle size, which may lead to segregation during dry blending and subsequent feeding of the material into the extruder. Within the QbD approach, the homogeneity of the initial blend was considered an important technological factor affecting the reproducibility of the composition and functional properties of the resulting filament.

According to TGA, the residual mass at 600 °C in seven dry-blend samples ranged from 18.66 to 22.41 wt.% (Table 5). The mean value was 20.06 ± 1.33 wt.% with an RSD of 6.64%. This value was close to the nominal mineral filler content of 20 wt.%, indicating the absence of pronounced macroscopic segregation of the mineral phase after dry blending.

The gentamicin sulfate content, determined spectrophotometrically after extraction from individual dry-blend samples, ranged from 4.682 to 5.470 wt.%. The mean value was 5.010 ± 0.291 wt.% with an RSD of 5.81%, which was very close to the nominal gentamicin sulfate content of 5 wt.%.

Thus, the TGA and spectrophotometric results confirm the acceptable compositional homogeneity of the dry PLA–Gen–MS blend prior to melt extrusion with respect to both the mineral filler and gentamicin sulfate.

### 3.2. Filament Production, Macroscopic Characterization, and Compositional Homogeneity

Since the processing temperature and extrusion conditions had previously been identified as critical process parameters (CPPs), their selection was considered one of the key factors affecting the quality and reproducibility of the resulting material. The main extrusion parameters and macroscopic characteristics of the obtained filament are presented in Table 6.

The most stable formation of the composite filament was observed at an extrusion temperature of 172 °C and a screw rotation speed of 2 rpm. Under these conditions, a continuous filament with a mean diameter of 1.75 ± 0.05 mm was obtained. The diameter remained within the specified tolerance along the length of the extruded material, and no pronounced macroscopic defects, such as cracks, delamination, or loss of integrity, were observed.

Macroscopically, the obtained material was a light-gray composite filament that retained its integrity during winding and subsequent handling. Representative macrophotographs of the obtained filament are presented in the Supplementary Materials (Figure S2). These results confirm that the selected composition enables the production of a continuous filament with a standard diameter, even with the simultaneous incorporation of a biogenic mineral filler and gentamicin, which is a necessary prerequisite for subsequent FDM processing and functional evaluation of the material. Thus, the selected processing conditions provided practical confirmation of stable filament formation and its geometric suitability for FDM printing.

The compositional homogeneity of the obtained PLA–Gen–MS filament along its length was additionally evaluated. This assessment was necessary because, even when the dry blend shows satisfactory homogeneity, the stages of material feeding, melting, and passage through the extruder may lead to local fluctuations in the content of the mineral filler and gentamicin sulfate. For this purpose, seven filament segments collected from different regions of the extruded material were analyzed.

The summarized results of the filament compositional homogeneity assessment are presented in Table 7. The residual mass at 600 °C was used as a compositional indicator of the thermally stable mineral-associated phase, whereas the gentamicin sulfate content was determined spectrophotometrically after antibiotic extraction. In this section, the residual mass at 600 °C was used specifically to evaluate compositional reproducibility; the detailed thermal behavior of the components and the composite is discussed separately in the thermogravimetric analysis section.

The residual mass at 600 °C in the seven filament segments ranged from 18.87 to 23.89 wt.%. The mean value was 21.72 ± 2.00 wt.% with an RSD of 9.19%. This value was close to the residual mass determined for the target PLA–Gen–MS filament during the overall TGA and indicates acceptable homogeneity of the thermally stable mineral phase along the filament length.

The gentamicin sulfate content in the seven filament segments ranged from 4.778 to 5.118 wt.%. The mean value was 4.963 ± 0.123 wt.% with an RSD of 2.49%. The closeness of the mean value to the nominal gentamicin sulfate content of 5 wt.% and the low RSD confirm the homogeneous distribution of the antibacterial component in the extruded filament.

Thus, the selected extrusion conditions enabled the production of a continuous filament with a standard diameter, while segmental analysis confirmed acceptable compositional homogeneity of PLA–Gen–MS along its length with respect to both the mineral component and gentamicin sulfate. These data support the reproducibility of the technological process and justify the subsequent evaluation of the structural, antibacterial, ion-releasing, and biological properties of the material.

### 3.3. Morphology, Elemental Distribution, and Quantitative Assessment of the Mineral Phase in the PLA–Gen–MS Filament

**Figure 3:**
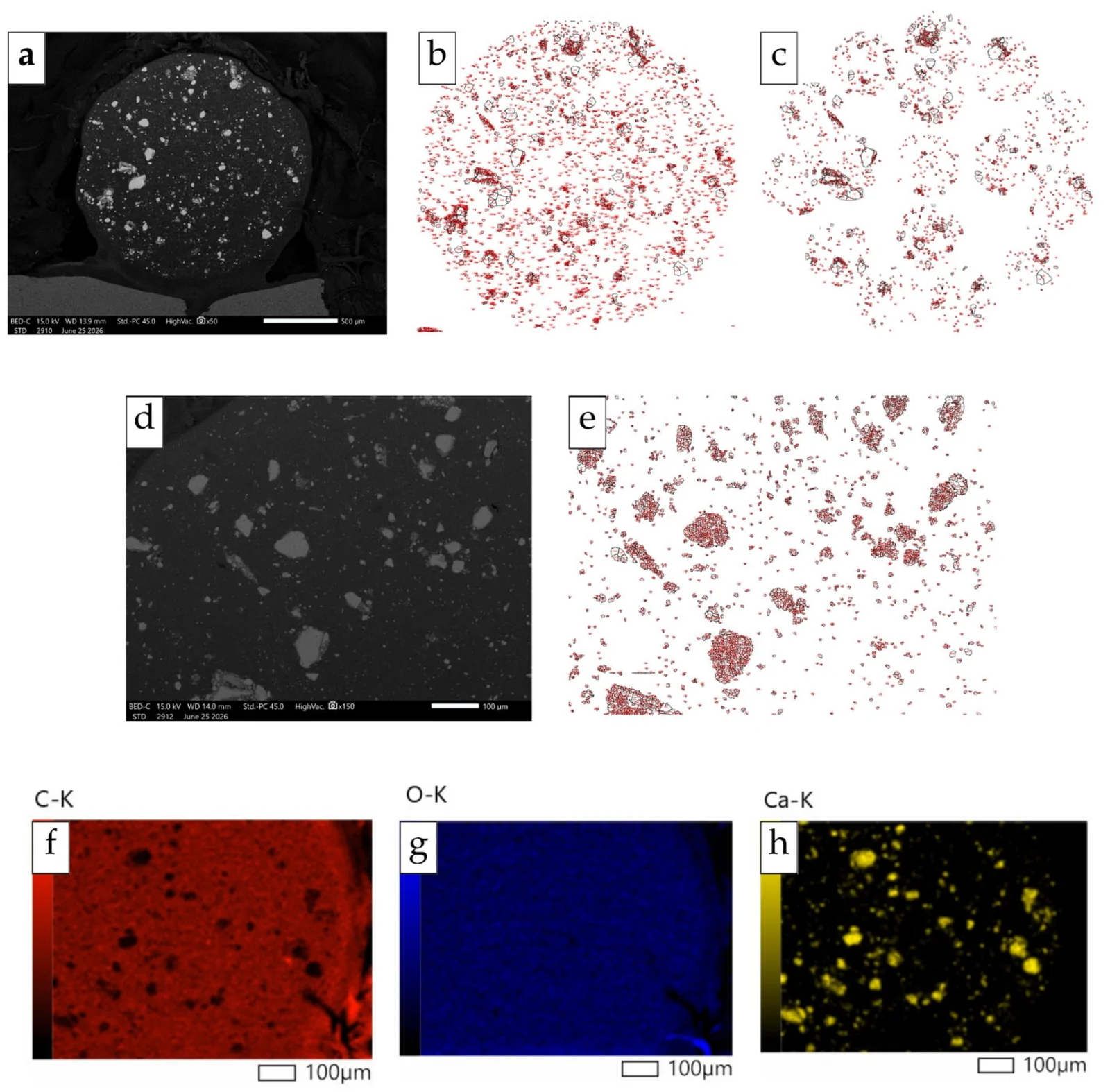
SEM–EDS characterization and ImageJ segmentation of the PLA–Gen–MS composite filament cross-section. (**a**) Overview BEC-SEM image of the filament cross-section; (**b**,**c**) ImageJ segmentation of mineral inclusions in the whole cross-section; (**d**) higher-magnification BEC-SEM image showing polydisperse mineral inclusions; (**e**) corresponding ImageJ segmentation of the higher-magnification image; (**f**–**h**) elemental distribution maps showing the spatial distribution of (**f**) C Kα—red, (**g**) O Kα—blue, and (**h**) Ca Kα—yellow. The colors correspond to element-specific EDS signals and do not represent separate chemical compounds.

The morphology of the PLA–Gen–MS filament after melt extrusion was examined by scanning electron microscopy using cross-sections of the samples at different magnifications (Figure 3). This approach allowed the general filament geometry, spatial distribution of the mineral phase, and features of its size organization to be systematically characterized.

Overview BEC images showed that the filament cross-section had an approximately circular shape and consisted of a compact, continuous PLA matrix without pronounced macroscopic defects, such as large pores, cracks, delamination, or disruption of material continuity (Figure 3a). Bright inclusions, corresponding to a phase with a higher mean atomic number, were observed in both central and peripheral regions of the cross-section. The mineral phase was not concentrated in a single localized region of the cross-section, indicating the absence of pronounced macroscopic segregation of the filler after dry blending and melt extrusion.

At ×150 magnification, the mineral phase exhibited a clearly polydisperse organization (Figure 3d). In addition to individual larger particles and small local clusters of shell-derived material, numerous fine inclusions were observed between larger mineral particles. Additional segmentation of the corresponding BEC image in ImageJ showed that some larger bright regions, after thresholding and Watershed separation, were segmented as groups of touching or closely spaced objects (Figure 3e). This indicates the clustered character of some mineral inclusions and explains the increase in the number of detected objects when the minimum particle-area threshold was reduced. Thus, the filler was represented by a wide size range and was distributed within the PLA matrix as a combination of individual larger inclusions, local clusters, and a numerous fine mineral fraction.

SEM–EDS analysis confirmed the calcium-containing nature of the bright inclusions (Figure 3f–h). The carbon map predominantly corresponded to the polymer matrix, whereas oxygen was present in both the polyester matrix and the mineral phase. The calcium signal spatially corresponded to the bright regions observed in the BEC images, confirming that these areas represented a calcium-containing mineral phase of shell origin. Taken together, the BEC-SEM and SEM–EDS data confirm the presence of CaCO_3_-containing filler in the structure of the extruded filament.

Nitrogen was also considered during EDS analysis because of its presence in the gentamicin structure; however, no distinct N signal was sufficiently resolved under the applied acquisition conditions for reliable elemental mapping. Therefore, nitrogen was not used as a marker of gentamicin distribution. Gentamicin content and compositional homogeneity were assessed independently by quantitative spectrophotometric analysis, as described in Section 2.4.

For quantitative assessment of the mineral phase, SEM images were processed in ImageJ using a fixed binarization protocol (Figure 3b,c,e). The main quantitative parameters of mineral-phase distribution are summarized in Table 8. Analysis of the whole cross-section identified 3105 mineral objects, with a total area corresponding to 6.752% of the analyzed cross-sectional area. The median object area (D50) was 16.731 µm^2^, indicating the numerical predominance of the fine mineral fraction in the presence of a smaller number of larger particles and local clusters.

Additional sensitivity analysis with respect to the minimum area of counted objects confirmed the pronounced polydispersity of the mineral phase. When the minimum particle-area threshold was reduced from 20 to 1 µm^2^, the number of detected objects increased approximately 22-fold. At the same time, the mineral area fraction changed much less markedly, from 6.089 to 8.408%. This confirms that small mineral objects substantially affect the particle count but make a limited contribution to the total filler area. This observation is consistent with higher-magnification BEC images, where some larger bright regions were partially represented by local clusters of smaller mineral objects.

To assess the spatial distribution of the filler, the cross-section was additionally analyzed using 16 equal-area circular regions of interest (ROIs) positioned in different regions of the section. The mean ROI area was 68201.455 µm^2^, with a coefficient of variation of 0.004%, confirming the nearly identical size of the analyzed regions. Mineral particles were detected in all ROIs; thus, no regions without detectable mineral inclusions were identified.

The local mineral area fraction varied from 3.198 to 21.684%, indicating microscale heterogeneity associated mainly with the presence of individual larger particles and local clusters. At the same time, D10 and D50 values within the ROIs were more stable, confirming the presence of the fine mineral fraction in all analyzed regions of the cross-section.

It should be noted that SEM/ImageJ analysis and TGA characterize different levels of material organization. TGA reflects an averaged compositional parameter for a sample mass or filament segment, whereas SEM/ImageJ describes the two-dimensional distribution of the visible mineral phase in a specific cross-section.

Overall, SEM at different magnifications, EDS mapping, and quantitative image analysis show that melt extrusion produced a compact PLA–Gen–MS filament containing a polydispersely distributed calcium-containing mineral phase. Larger particles and small local clusters determined local maxima in filler area, whereas the numerous fine mineral fractions were distributed throughout the cross-section. At the level of the whole cross-section, no signs of pronounced macroscopic filler segregation were observed, while local variability was mainly associated with the polydisperse and clustered character of the biogenic mineral filler.

### 3.4. Thermogravimetric Analysis and Thermal Stability of the Target PLA–Gen–MS Filament

**Figure 4:**
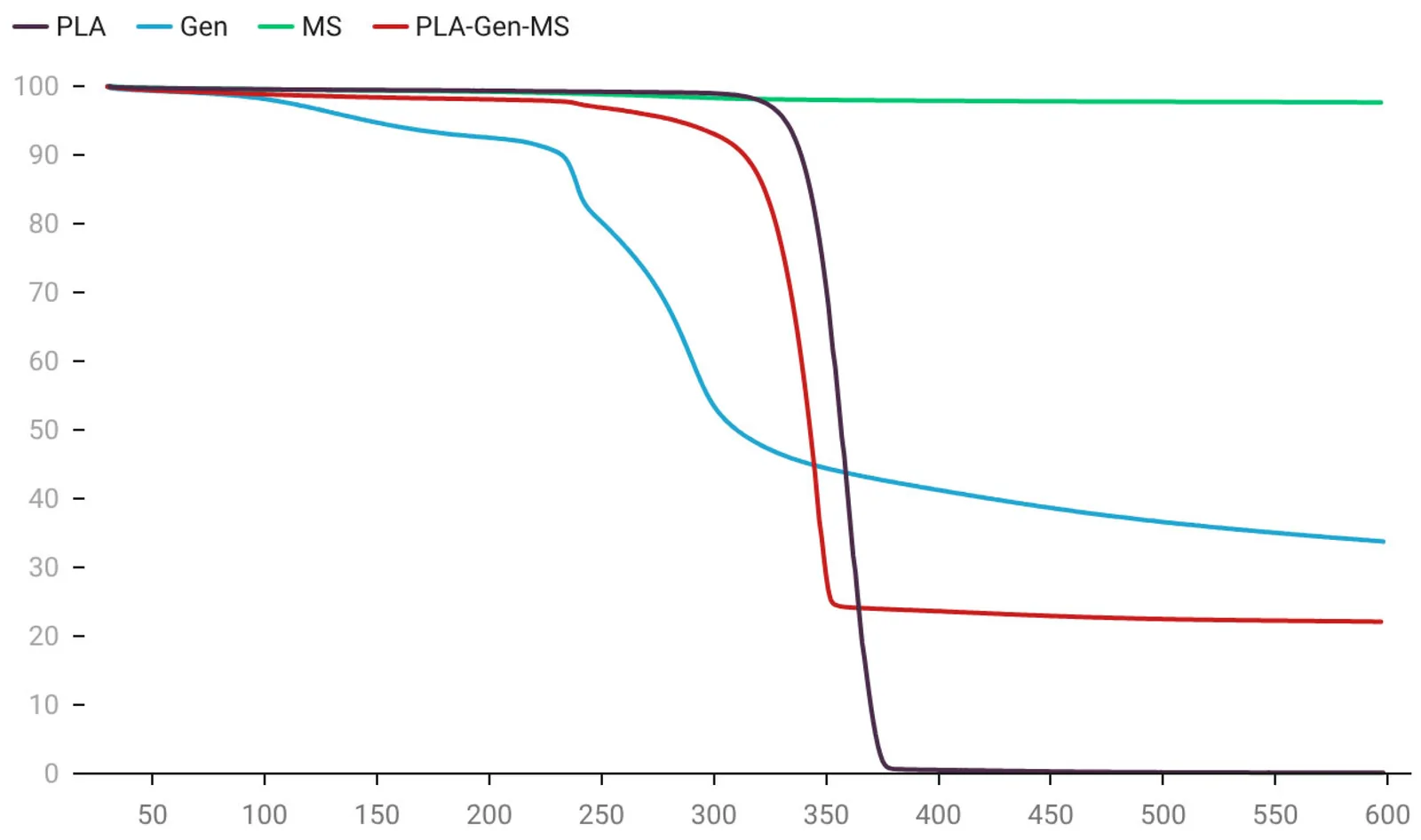
TG curves of individual components and the target PLA–Gen–MS composite filament: PLA, gentamicin (Gen), mollusk shell filler (MS), and PLA–Gen–MS composite.

Considering that thermal stability was previously identified as a critical quality attribute, while extrusion temperature was identified as a critical process parameter, thermogravimetric analysis was used to assess the compatibility of the components with melt-processing conditions and to confirm the thermal stability of the target composite. TG curves for PLA, gentamicin, mollusk shell filler, and the target PLA–Gen–MS composite filament are presented in Figure 4, and the main thermal parameters calculated from the TGA curves are summarized in Table 9.

Neat polylactic acid exhibited typical single-stage thermal decomposition, with high stability before the onset of intensive degradation. The Tonset, T5%, and Tmax values were 342.5 °C, 331.5 °C, and 358.8 °C, respectively, with nearly complete mass loss by 600 °C and a residual mass of 0.11%. The mollusk shell filler showed high thermal stability throughout the investigated temperature range: mass loss did not exceed 3%, and the residual mass at 600 °C was 97.61%, indicating the predominance of a thermally stable mineral phase.

Gentamicin showed multistage thermal behavior. In the low-temperature region, mass loss was observed, likely associated with the removal of physically and structurally bound water, whereas the main degradation of the organic structure occurred at higher temperatures (Tonset 230.5 °C, Tmax 239.0 °C) and was accompanied by the formation of a carbonaceous residue under an inert atmosphere.

The thermogravimetric profile of the PLA–Gen–MS composite filament reflected the combined contribution of all system components. The main mass loss occurred within the range of 280–360 °C and was mainly associated with decomposition of the PLA matrix. The Tonset, T5%, and Tmax values for the composite (328.9 °C, 283.3 °C, and 345.3 °C, respectively) were lower than those of neat PLA, which may be related to the presence of functional additives and their effect on the thermal degradation behavior of the polymer matrix. Nevertheless, the shape of the TG curve and the overall decomposition temperature range remained close to those of PLA, indicating no abrupt change in the thermal degradation profile of the polymer.

The residual mass of the composite at 600 °C was 22.08%, which is in good agreement with the nominal mineral phase content in the material formulation (20 wt.%) and may additionally reflect the contribution of the carbonaceous residue formed during gentamicin thermal decomposition. Taken together, these findings indirectly confirm the consistency of the filament composition with the target formulation and the presence of a thermally stable mineral component in the system.

Importantly, the processing temperature used for filament extrusion (172 °C) was substantially lower than the temperatures corresponding to the onset of intensive degradation of both the PLA matrix and the composite as a whole. Therefore, the obtained data indicate sufficient thermal compatibility of the components under short-term melt-processing conditions and confirm the suitability of the target material for extrusion, consistent with the CQA of thermal stability.

### 3.5. Differential Scanning Calorimetry of the Target PLA–Gen–MS Filament

**Figure 5:**
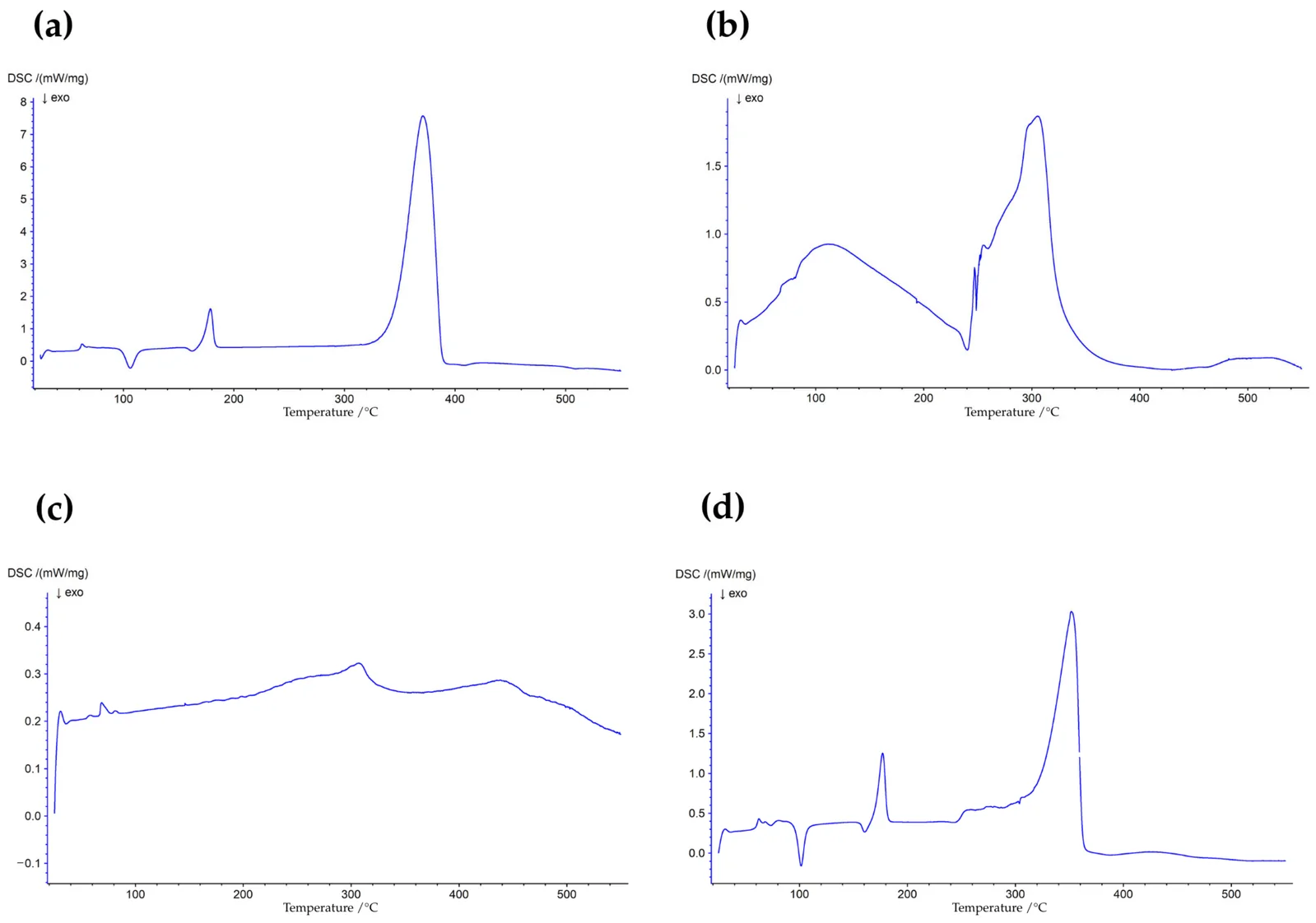
DSC curves of individual components and the target PLA–Gen–MS composite filament: (**a**) PLA, (**b**) gentamicin, (**c**) mollusk shell filler (MS), and (**d**) PLA–Gen–MS. The downward arrows (↓) indicate the exothermic direction.

In addition to resistance to thermal degradation, it was important to determine whether the PLA matrix retained its characteristic thermal transitions after incorporation of the biogenic mineral phase and gentamicin. For this purpose, DSC analysis was performed for the individual components and the PLA–Gen–MS filament. The DSC curves are presented in Figure 5, and the main thermal transitions identified from the DSC curves are summarized in Table 10.

Neat polylactic acid exhibited thermal transitions typical of PLA, including glass transition (Tg ~60 °C), cold crystallization (Tc ~106 °C), and melting (Tm ~179 °C). In the target PLA–Gen–MS composite, these transitions were also retained, with Tg, Tc, and Tm values of approximately ~60 °C, ~102 °C, and ~177 °C, respectively. Compared with neat PLA, the composite showed a slight shift in the cold crystallization and melting temperatures toward lower values, while the overall structure of the DSC profile was preserved. This indicates that the incorporation of mollusk shell filler and gentamicin does not disrupt the basic thermal behavior of the PLA matrix, although it affects specific features of its crystallization behavior.

The mollusk shell powder did not show pronounced endothermic or exothermic effects in the analyzed region (Figure 5c), which is consistent with the predominance of a thermally stable mineral phase. Gentamicin was characterized by the absence of a clearly defined melting peak (Figure 5b); the observed broad endothermic effect in the low-temperature region may be associated with the removal of bound water.

Thus, the DSC results demonstrate that functionalization of PLA with the biogenic mineral phase and gentamicin does not lead to the loss of the characteristic thermal transitions of the polymer matrix. Together with the TGA data, these findings confirm the thermal compatibility of the components and preservation of composite processability under melt-extrusion conditions.

### 3.6. Mechanical Properties of the PLA–Gen–MS Filament

The mechanical properties of the target PLA–Gen–MS composite were evaluated based on tensile strength and flexural strength. The test results are presented in Table 11.

Compared with the original PLA matrix, the incorporation of the biogenic mineral filler and gentamicin resulted in reduced mechanical strength. Tensile strength decreased from 50.91 ± 3.79 MPa for neat PLA to 30.06 ± 0.93 MPa for PLA–Gen–MS. A similar trend was observed in flexural testing, where flexural strength decreased from 113.30 ± 1.67 MPa to 41.63 ± 6.71 MPa.

Despite the decrease in strength parameters, the composite retained mechanical integrity and was suitable for filament handling and subsequent FDM processing. These data indicate that the incorporation of functional components reduced the strength compared with neat PLA, but did not compromise the mechanical integrity required for filament handling and fabrication of model scaffolds by FDM printing.

### 3.7. Ion Profile of the PLA–Gen–MS Aqueous Extract Determined by ICP-AES

The elemental composition of the aqueous extract of the PLA–Gen–MS composite, prepared with reference to ISO 10993-12:2021 [51], was analyzed by ICP-AES. The quantitative results are presented in Table 12.

As shown in Table 12, the ionic profile of the extract was distinctly calcium-dominant. The calcium concentration was 284 mg/dm^3^, exceeding the concentrations of most other detected elements by several orders of magnitude.

Based on the experimentally determined calcium content of the mollusk shell-derived mineral phase (396,067 mg/kg) and its 20 wt.% loading in the composite, the initial calcium content of PLA–Gen–MS was estimated at approximately 79.21 mg/g. At the material-to-extraction-medium ratio used in this study (1 g/10 mL), the measured calcium concentration of 284 mg/dm^3^ corresponded to approximately 2.84 mg of Ca released per gram of composite after 72 h. Accordingly, approximately 3.59% of the initial calcium content was released, while approximately 96.41% was estimated to remain within the composite. The remaining fraction was calculated by mass balance and was not determined by direct elemental analysis of the extracted composite.

In addition to calcium, measurable amounts of Sr (1.15 mg/dm^3^), P (0.84 mg/dm^3^), Mg (0.60 mg/dm^3^), Mn (0.420 mg/dm^3^), and Fe (0.25 mg/dm^3^) were detected in the extract. This combination of elements reflects the biogenic nature of the mollusk shell filler and indicates its ability to serve not only as a structural component but also as a functional phase within the composite.

Other elements (B, Ba, Al, Zn, Cu, Cr, Ce, Ni, V, Co, Mo, and La) were present at substantially lower concentrations (<0.1 mg/dm^3^), whereas the concentrations of the remaining analyzed elements were below the limit of detection (LOD). Thus, the release profile was characterized by the formation of a multicomponent ionic microenvironment with a dominant calcium contribution.

**Figure 6:**
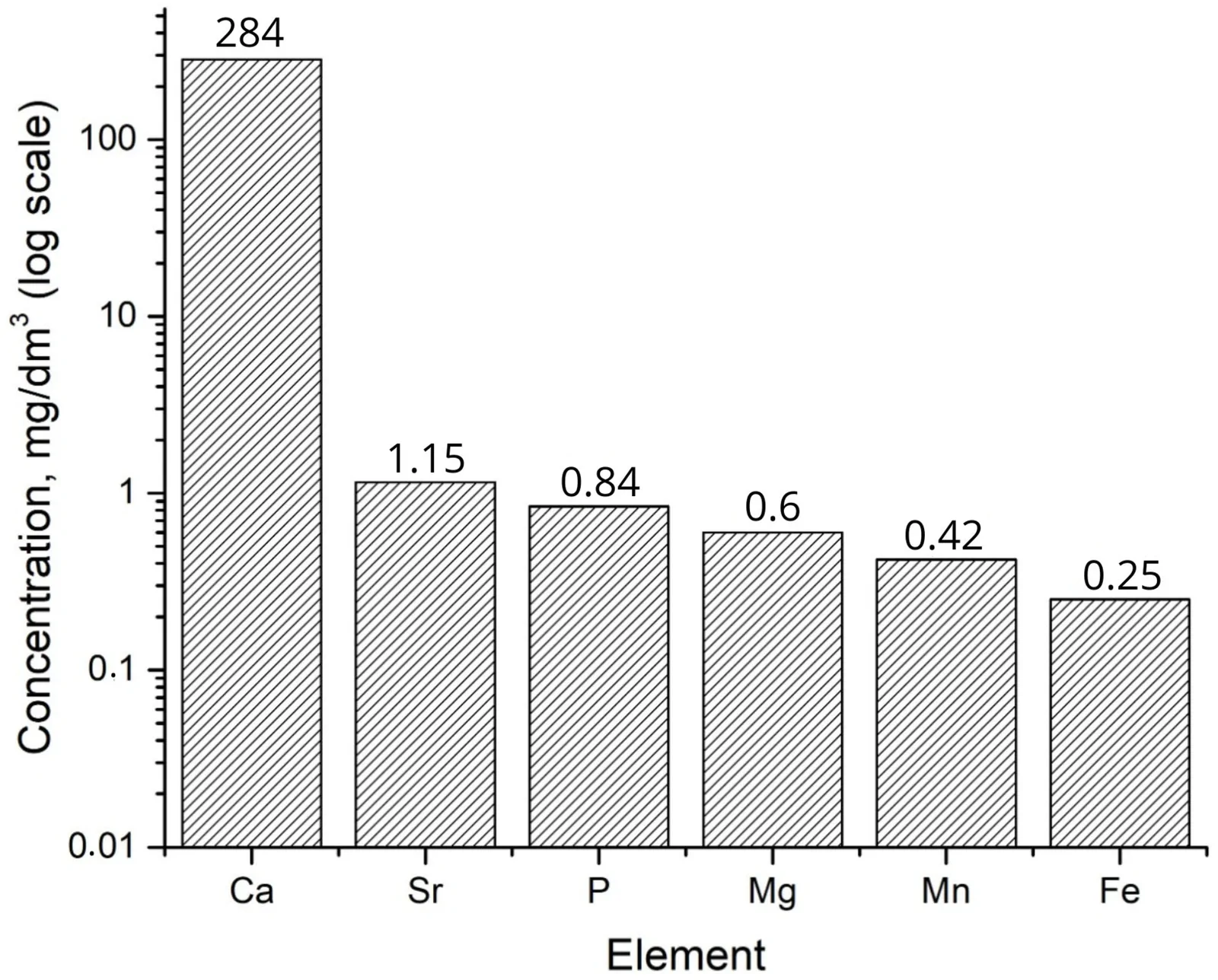
Log-scale visualization of the ion release profile from PLA–Gen–MS based on ICP-AES data.

For visual representation of the ionic profile, the concentrations of the main elements were plotted as a bar chart with a logarithmic concentration axis (Figure 6).

The data presented in Figure 6 further emphasize the hierarchical nature of the observed profile: calcium markedly predominates over all other components, whereas strontium, phosphorus, and magnesium form a group of measurable but substantially less concentrated ions. Manganese and iron are present at trace but detectable levels. The use of a logarithmic scale enables clear comparison of elements whose concentrations differ by several orders of magnitude.

Thus, the ICP-AES results show that the PLA–Gen–MS composite should not be regarded as an entirely chemically inert system, but rather as a calcium-dominant ion-releasing material containing additional trace elements of biogenic origin. From a QbD perspective, this confirms the ability of the material to generate the intended ionic profile and reinforces the interpretation of the mollusk shell filler as a functional rather than merely structural phase.

### 3.8. Antibacterial Activity of the PLA–Gen–MS Composite (Agar Diffusion Test)

The antibacterial activity of the target PLA–Gen–MS composite was evaluated using the agar diffusion test with standard bacterial strains. Quantitative results, expressed as inhibition zone diameters, are presented in Table 13. Representative Petri dish images obtained during the agar diffusion assay are provided in the Supplementary Materials (Figure S3).

As shown in Table 13, the PLA–Gen–MS composite demonstrated pronounced antibacterial activity against all tested bacterial strains. The inhibition zone diameters were 20.3 ± 0.9 mm for *Staphylococcus aureus* ATCC 6538, 21.0 ± 0.6 mm for *Escherichia coli* ATCC 25922, and 21.0 ± 1.0 mm for *Pseudomonas aeruginosa* ATCC 27853.

Comparison with control samples indicated that the observed effect was attributable to the presence of gentamicin in the composite. As antibiotic-free controls, pure PLA and PLA–MS were included to distinguish the intrinsic response of the PLA matrix and the mineralized PLA matrix from the gentamicin-containing PLA–Gen–MS composite. Both antibiotic-free controls produced only low values close to the specimen/contact diameter. PLA and PLA–MS showed no measurable inhibition beyond the specimen edge: the recorded values were minimal (7.0–8.0 mm) and mainly corresponded to the direct contact zone around the sample. Therefore, the values recorded for PLA and PLA–MS should not be interpreted as intrinsic antibacterial activity. Such weak apparent clear/contact zones may occur in agar diffusion assays with polymer-based specimens and may reflect local interfacial effects at the material–agar boundary, including minor pH shifts associated with initial PLA hydrolysis or local changes in hydration and moisture conditions. The positive control—gentamicin sulfate—produced inhibition zones of 15.3 ± 0.9 mm for *S. aureus*, 15.7 ± 0.3 mm for *E. coli*, and 20.6 ± 1.2 mm for *P. aeruginosa*. The activity of PLA–Gen–MS was comparable to that of the gentamicin disk under the applied agar diffusion conditions.

Thus, the results of the agar diffusion test confirm that the PLA–Gen–MS composite retains antibacterial functionality after incorporation of gentamicin and melt processing. Within the QbD framework, these findings confirm the successful achievement of a key quality attribute related to antibacterial activity.

### 3.9. Preliminary In Vivo Biocompatibility Assessment and Tissue Response

**Figure 7:**
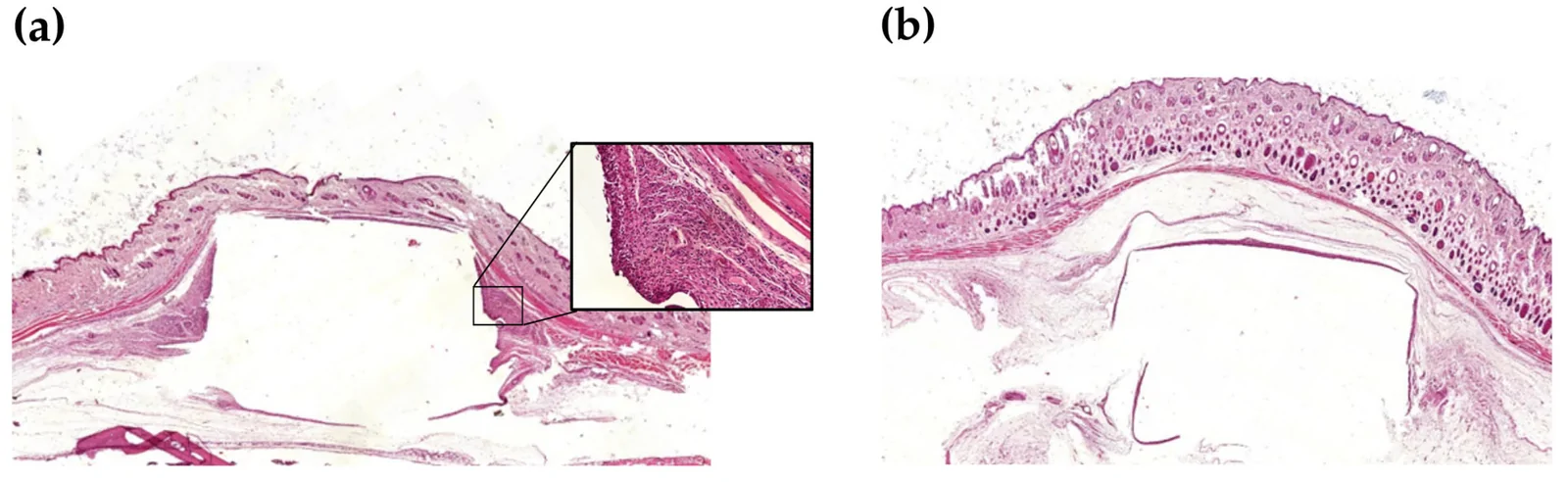
Representative histological images of tissue response after subcutaneous implantation. (**a**) TiLOOP^®^ reference implant; (**b**) PLA–Gen–MS composite.

A key step in validating PLA–Gen–MS as a material for implant applications was the assessment of its in vivo biocompatibility. Within the QbD framework, this stage was considered a verification of a critical quality attribute related to material biocompatibility. The study was conducted using a subcutaneous implantation model in rats, with a commercially available titanium-coated polypropylene implant, TiLOOP^®^, used as the reference group. Morphometric and semi-quantitative histological data are presented in Table 14 and Figure 7.

In the TiLOOP^®^ reference group, a pronounced inflammatory response was observed, with the maximum thickness of inflammatory infiltration on Day 3 (2468.3 μm), followed by a gradual decrease by Day 14 (357.2 μm). Histologically, the early tissue response was characterized by infiltration with neutrophils and macrophages, formation of granulation tissue, and the presence of multinucleated foreign body giant cells, indicating a pronounced reaction to the implanted material.

In the PLA–Gen–MS group, the dynamics of tissue response were characterized by a more rapid reduction in inflammatory infiltration. By Day 7, the thickness of infiltration decreased to 749.2 μm and was significantly lower compared with the TiLOOP^®^ group (*p* < 0.05). By Day 14, inflammatory infiltration was no longer detected, and this parameter also differed significantly from TiLOOP^®^ (*p* < 0.05). No foreign body giant cells or granulomatous structures were observed in the PLA–Gen–MS group, and the tissue exhibited signs of remodeling.

Thus, the PLA–Gen–MS composite demonstrated a more favorable preliminary biocompatibility profile compared with the reference implant, as evidenced by the resolution of inflammatory infiltration by Day 14, absence of a pronounced foreign body reaction, and signs of tissue remodeling. Within the applied model, these findings confirm the achievement of the CQA related to biocompatibility and support further investigation of the material in the context of biomedical and implant applications.

### 3.10. Additive Processability and FDM Fabrication of Model Scaffolds

**Figure 8:**
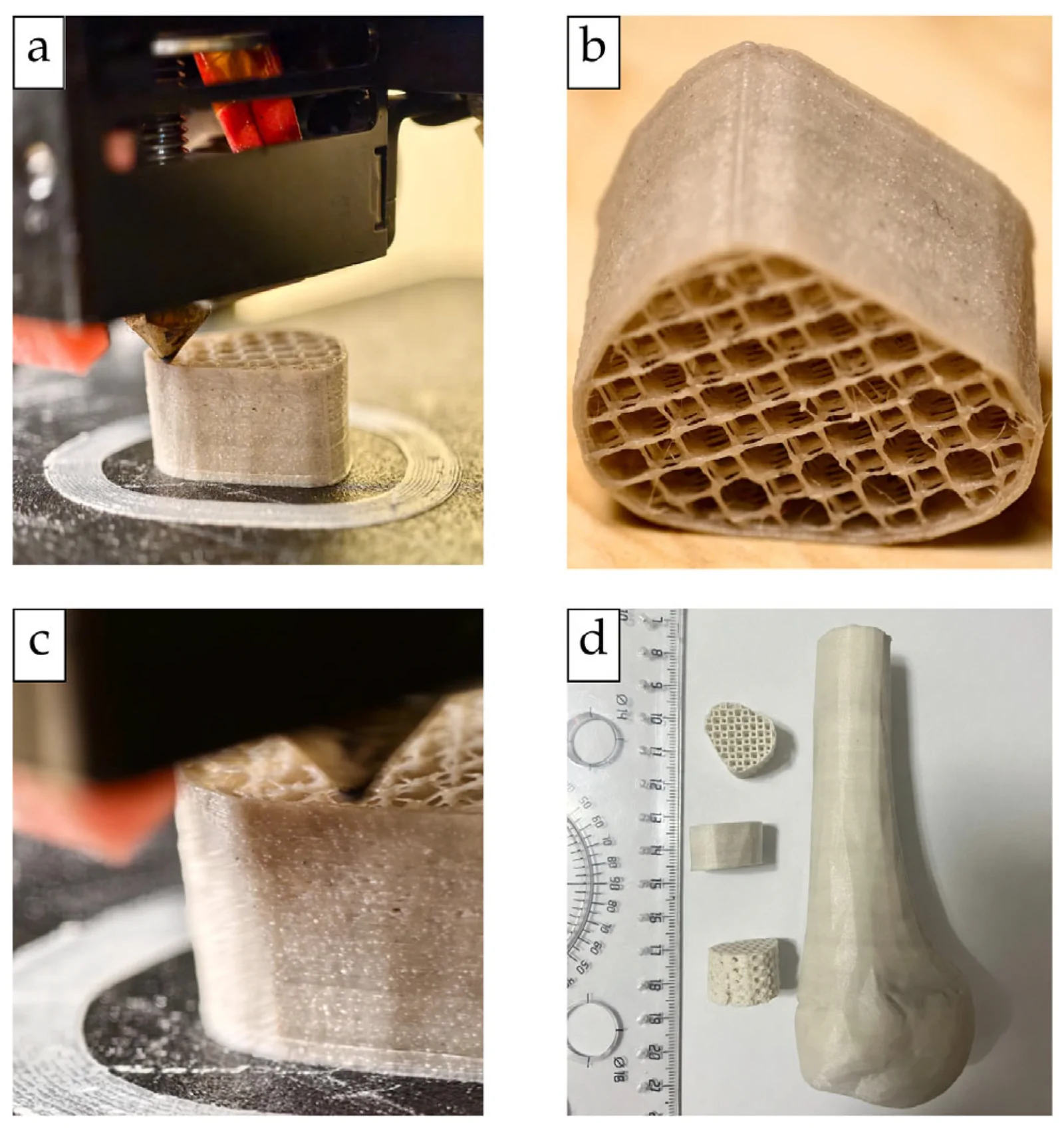
FDM printability of the PLA–Gen–MS filament and representative printed structures. (**a**) Continuous filament deposition during printing of the PLA–Gen–MS scaffold; (**b**) printed scaffold with regular lattice architecture; (**c**) magnified view of filament deposition during printing; (**d**) representative printed objects with different geometries, including lattice scaffolds and a model anatomical structure.

The suitability of the PLA–Gen–MS filament for FDM printing was confirmed by the fabrication of test specimens and model structures with different geometries. The printed structures shown in Figure 8 and Supplementary Video S1 were fabricated using the same PLA–Gen–MS filament composition described in Section 2.3 and Section 3.2. Within the QbD framework, this stage was considered a verification of the critical quality attribute related to printability and technological feasibility. The printing conditions and parameters used for the assessment are described in Section 2.12.

Continuous filament feeding and stable material deposition were maintained throughout the printing process. No nozzle clogging, filament breakage, or pronounced interlayer delamination was observed.

The filament enabled the fabrication of structures with different geometric complexity, including regular lattice scaffolds and a model anatomical structure (Figure 8). The lattice structures retained the intended overall architecture, with continuous deposited strands and visually distinguishable interconnected pores. Successful fabrication of the more geometrically complex model further demonstrated that the composite filament could be processed beyond simple test geometries without loss of continuous material deposition.

A representative video recording of the FDM process is provided in the Supplementary Materials (Video S1).

Overall, the absence of major processing interruptions, together with successful fabrication of both regular lattice structures and a more complex model geometry, confirmed the FDM processability of the PLA–Gen–MS filament under the selected processing conditions. These results support achievement of the CQA related to filament printability and demonstrate the technological feasibility of using the developed composition for additive manufacturing of model biomedical constructs.

## 4. Discussion

Despite the rapid development of materials for additive manufacturing in bone tissue engineering, most existing PLA-based composites remain functionally fragmented. Some systems are primarily designed to enhance osteoconductivity through the incorporation of hydroxyapatite, bioactive glass, or other calcium-containing fillers [11,13,21], whereas others focus on imparting antimicrobial properties via surface modification or the incorporation of specific antibacterial agents [17,54]. While such approaches allow improvement of individual material characteristics, they do not provide the integrated functional response required for implantable constructs, where processability, structural integrity, bioactivity, localized antimicrobial protection, and biocompatibility must be achieved simultaneously [11,54].

In the present study, an alternative strategy is proposed and experimentally validated: the development of a QbD-designed multifunctional filament platform in which a biodegradable PLA matrix, a biogenic calcium-containing filler derived from mollusk shells, and gentamicin are integrated into a single thermoplastic composite system. The key novelty of this work lies not only in the material composition but also in the underlying design logic. In contrast to empirical formulation approaches [55], the Quality by Design (QbD) framework [30,56] enables the prior definition of the target product profile, critical quality attributes, and the most significant compositional and process parameters. It should be noted that the application of QbD to the development of thermoplastic filaments for FDM printing remains limited in the literature [57], whereas this approach is more commonly applied in pharmaceutical development [30,56,58] and in selected biomaterial systems. In this context, the present study represents a systematic approach to the design of a multifunctional PLA filament within the «composition–processing–structure–function» paradigm [55,57].

The selection of the mineral phase plays a critical role in the proposed strategy. In most studies, synthetic calcium-containing materials such as hydroxyapatite or bioactive glass are traditionally used to modify PLA [11,21,59]. Despite their widespread application, such fillers typically provide predominantly osteoconductive effects associated with apatite formation, increased surface hydrophilicity, and support of cell adhesion, while their ionic profile is generally defined by the intrinsic composition of the filler [11,13].

In contrast, mollusk shells represent a biogenic mineral–organic phase in which calcium carbonate is the dominant mineral component, together with a minor organic matrix and associated macro- and trace elements, including Mg, Sr, P, Mn, and Fe [60,61]. Previous studies have reported that mollusk shells consist predominantly of CaCO_3_, commonly exceeding 90% by mass, whereas XRD/FTIR-based characterization of shell-derived materials confirms that this CaCO_3_ phase is mainly represented by aragonite and/or calcite polymorphs [62,63]. Therefore, the mineral phase introduced into PLA–Gen–MS should be interpreted primarily as a shell-derived CaCO_3_-based filler rather than as a hydroxyapatite- or tricalcium phosphate-based filler. This distinction is important because PLA–Gen–MS is not positioned as a preformed calcium phosphate bone substitute, but as a multifunctional PLA-based filament platform in which the expected biological contribution of the mineral phase is associated with calcium-dominant ion release, carbonate mineral reactivity, and biologically relevant trace elements.

Consistent with this mineralogical context, ICP-AES confirmed that PLA–Gen–MS generated a calcium-dominant yet multicomponent ionic profile, with measurable levels of Sr, Mg, P, Mn, and Fe. In carbonate polymorphs, Sr^2+^ and Mg^2+^ may be incorporated into the CaCO_3_ structure in different ways. XAFS studies have shown that Sr^2+^ can substitute almost ideally for Ca^2+^ in aragonite and can also occupy Ca sites in calcite, although with greater local structural distortion; in contrast, Mg incorporation is more complex and may involve local structural relaxation or Mg-rich carbonate-associated domains [64]. Therefore, Sr and Mg detected in the PLA–Gen–MS extract are most plausibly associated with the carbonate mineral phase inherited from the mollusk shell filler. P was detected only as a minor released element and should not be interpreted as evidence of a dominant calcium phosphate phase; rather, it may be associated with minor phosphate-containing species, trace mineral impurities, or the organic matrix of the biogenic filler. Together, these findings support the interpretation of PLA–Gen–MS as a CaCO_3_-based biogenic mineral system capable of generating a calcium-dominant, multicomponent ionic microenvironment.

Considering the established roles of Ca^2+^ in mineralization and cell adhesion, Mg^2+^ in the regulation of osteogenic differentiation, angiogenesis, and cell–matrix interactions, as well as Sr^2+^ in stimulating osteogenic differentiation and reducing osteoclastic activity [60,65], this ion-release profile suggests that the biogenic filler may contribute not only to the structural organization of the composite, but also to the formation of a potentially bioactive ionic microenvironment.

However, the functional potential of the mineral phase can only be realized through its controlled integration into the polymer matrix while maintaining the processability of the composite [55]. For this reason, during the risk assessment stage, PLA molecular weight, mineral filler content and dispersion, gentamicin loading, extrusion temperature, and residence time in the heating zone were identified as factors potentially affecting filament quality. Experimental results confirmed the relevance of this QbD-based rationale: the selected processing parameters enabled the production of a continuous filament with a diameter of 1.75 ± 0.05 mm, suitable for subsequent FDM printing.

Importantly, the QbD-oriented processing strategy was further supported by a two-stage assessment of compositional homogeneity before and after extrusion. In the dry PLA–Gen–MS blend, the residual mass at 600 °C, used as a compositional indicator of the thermally stable mineral-associated fraction, was close to the nominal content of the mineral filler, whereas spectrophotometric analysis showed a gentamicin sulfate content close to the target value of 5 wt.%. After melt extrusion, segmental analysis of the filament confirmed acceptable reproducibility of the mineral-associated residue along the filament length and high homogeneity of gentamicin distribution. These results indicate that the selected dry-mixing and extrusion conditions did not lead to pronounced compositional segregation of either the mineral phase or the antibacterial component.

Thus, in the present work, QbD functions not only as a planning framework but also as a tool for predictive material design, linking the intrinsic properties of the components, processing parameters, and the target functional properties of the filament [30,56]. In this regard, the proposed strategy follows a broader paradigm of controlled technological processes, in which processing conditions, exposure time, and environmental parameters are considered key factors determining the reproducibility of the final outcome [66].

This relationship was supported by the results of morphological, compositional, thermal, and mechanical analyses. SEM–EDS and ImageJ analysis showed that the mineral phase was present throughout the entire cross-section of the PLA matrix, without the formation of a single filler-enriched zone, extended cracks, or pronounced interfacial debonding. At the same time, quantitative image analysis revealed a polydisperse mineral architecture, in which numerous finely dispersed inclusions coexisted with larger particles and local accumulations. Therefore, the microstructure of PLA–Gen–MS should be interpreted not as ideally homogeneous, but as macroscopically non-segregated and locally microheterogeneous, which is expected for composites containing a natural polydisperse mineral filler. Such a microstructure is critically important because the quality of filler dispersion and the state of the interfacial boundary substantially influence the printability, mechanical properties, and functional reproducibility of PLA composites [67]. Local filler accumulations may act as stress concentrators, reduce mechanical reliability, and potentially affect local diffusion pathways for the release of active components [67,68].

The absence of pronounced macroscopic segregation, preservation of PLA-matrix continuity, and acceptable compositional homogeneity according to TGA and spectrophotometric data are consistent with the technological integrity of the material during extrusion, filament handling, and subsequent printing. At the same time, the local microstructural variability revealed by SEM/ImageJ does not contradict the data on compositional reproducibility, since SEM characterizes the two-dimensional distribution of the mineral phase in an individual cross-section, whereas TGA and spectrophotometry reflect the averaged composition of a weighed sample or a filament segment.

**Figure 9:**
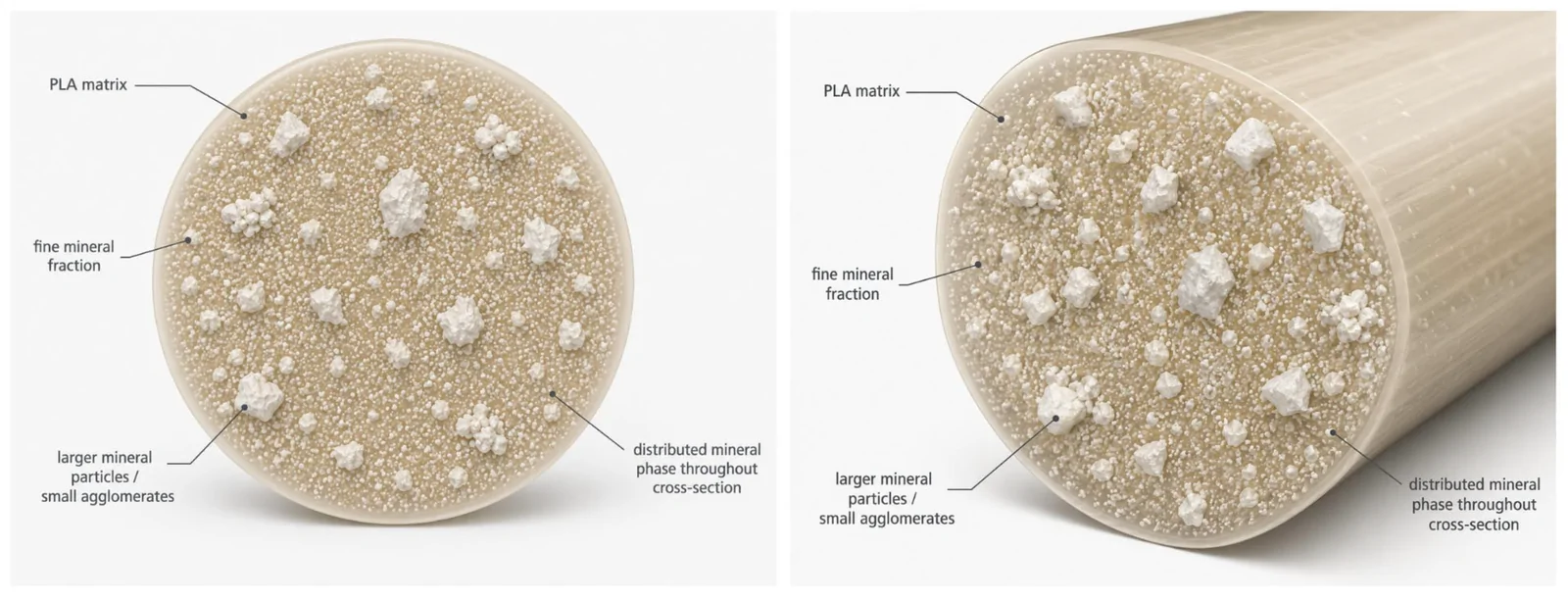
Conceptual AI-assisted 3D visualization of the PLA–Gen–MS filament microstructure. The visualization was generated with the assistance of ChatGPT (GPT-5.6 Sol; OpenAI, San Francisco, CA, USA) based on SEM/BEC imaging and ImageJ segmentation results. It does not represent a tomographic reconstruction or primary quantitative data.

To summarize the SEM/BEC observations and ImageJ segmentation data, a schematic 3D visualization of the internal organization of the PLA–Gen–MS filament was constructed (Figure 9).

This scheme is not a tomographic reconstruction, but rather an interpretative model based on the analysis of cross-sections. It reflects the two-level organization of the mineral phase: a combination of individual large CaCO_3_-containing inclusions or local accumulations with a numerous finely dispersed fraction distributed throughout the PLA matrix. Such a microstructure may increase the PLA/mineral-phase interfacial area and potentially influence the interaction of the composite with the aqueous environment, interfacial diffusion pathways, and the transport of hydrophilic molecules.

Thermal analysis in the present work served two complementary functions. First, TGA was used to assess the thermal stability of the components and the composite relative to the selected extrusion temperature. Second, the residual mass at 600 °C was used as a compositional indicator of the thermally stable mineral-associated fraction when evaluating the homogeneity of the dry blend and filament segments. This approach made it possible to link the thermal suitability of the material for processing with the control of compositional reproducibility. TGA showed that the main thermal degradation of PLA–Gen–MS occurred at temperatures substantially higher than the extrusion temperature, whereas DSC confirmed the preservation of the characteristic thermal transitions of PLA after incorporation of the mineral filler and gentamicin. Therefore, PLA functionalization was not accompanied by any significant disruption of the basic thermal organization of the polymer matrix.

This is particularly important because the system contains gentamicin, an antibiotic that may be sensitive to elevated temperatures. As shown above, the onset of the main thermal degradation of gentamicin occurred above the temperature range used for short-term material processing, which is consistent with literature data on the thermal stability of gentamicin sulfate [69]. The preservation of antibacterial activity after extrusion further confirms that the selected processing conditions did not lead to functionally significant thermal degradation of the antibiotic. In addition, spectrophotometric analysis of filament segments showed that the gentamicin sulfate content remained close to the nominal level of 5 wt.% with low variability along the filament length. Therefore, the antibacterial functionality of PLA–Gen–MS was confirmed not only by the preservation of microbiological activity after extrusion, but also by quantitative data demonstrating the homogeneity of gentamicin content in the final filament.

The selection of gentamicin was based on its combination of pharmacological relevance and technological suitability for the present filament system. Gentamicin is a broad-spectrum aminoglycoside with established use in orthopedic local antibiotic-delivery approaches, particularly antibiotic-loaded bone cements and related carrier-based systems [41,42]. In addition, its powder form, water solubility, and relative thermal stability make it suitable for incorporation into polymer-based matrices and short-term melt-processing conditions [41,69]. Accordingly, gentamicin was selected as a clinically relevant antibiotic component to validate the feasibility of producing an antibacterial PLA-based filament by solvent-free melt extrusion. Further development of such systems should include quantitative release studies, susceptibility-guided antibiotic selection, and assessment of resistance-selection risks associated with local antibiotic exposure [41,42].

Mechanical testing showed the expected decrease in the mechanical strength of PLA–Gen–MS compared with the original PLA matrix. This behavior is consistent with data reported for mineral-filled PLA and PLA/PCL composites, where an increase in the inorganic phase content may be accompanied by reduced strength due to the formation of a more heterogeneous structure, an increased number of interfacial boundaries, the presence of local filler accumulations, increased porosity, and reduced efficiency of mechanical load transfer within the material [70,71].

Importantly, the observed reduction in strength was not accompanied by a loss of filament structural integrity or deterioration of its processability in FDM printing. This indicates that the material retained its technological suitability despite modification of its composition.

It should be emphasized that the obtained mechanical characteristics must be interpreted in the context of the intended application of the developed composite. PLA–Gen–MS is not considered a material for load-bearing bone reconstruction, but rather is positioned as a bioactive and antibacterial filament platform for the fabrication of porous scaffolds and temporary implantable constructs. In this context, the key objective is not to maximize strength, but to achieve a functional balance between mechanical integrity, printability, Ion-releasing properties, and local antibacterial activity [72]. The flexural strength of PLA–Gen–MS, although lower than that of neat PLA, remains within a range that may be relevant for temporary scaffold-forming applications in low-load-bearing or anatomically protected bone defects. For comparison, the mechanical strength of trabecular bone is substantially lower than that of cortical bone and varies widely depending on anatomical location, density, and microarchitecture; Mohd Ghazali et al. reported an average three-point bending strength of approximately 17.0 MPa for cancellous bone [73]. In this context, the flexural strength of PLA–Gen–MS indicates its potential relevance as a temporary defect-filling or scaffold-forming material in low-load-bearing regions.

Thus, the decrease in mechanical properties in this case should be regarded as an expected trade-off associated with the expanded functionality of the material, rather than as a factor precluding its use as a bioactive filament platform. This balance between mechanical and biologically relevant properties is consistent with current approaches to the development of bioactive composites for regenerative medicine.

The functional validity of the proposed compositional and technological architecture is further supported by the results of antibacterial testing. PLA–Gen–MS demonstrated pronounced antimicrobial activity against all tested bacterial strains after gentamicin incorporation and subsequent melt processing of the composite. Previous studies have shown the fundamental feasibility of integrating antibiotics, including gentamicin, into a PLA matrix, followed by active agent release and preservation of antimicrobial activity after thermal processing [37,47]. However, those studies considered PLA–antibiotic systems without a biogenic mineral phase and without the formation of a three-component bioactive filament.

In the present study, this approach was extended to a more complex PLA–Gen–MS system, in which gentamicin was integrated into the bulk of the PLA matrix together with a biogenic calcium-containing filler. This is of independent significance because the mineral phase may influence antibiotic distribution, composite microstructure, diffusion pathways, and subsequent release behavior. Therefore, the preservation of antimicrobial activity in PLA–Gen–MS after extrusion confirms not only the reproducibility of the previously described effect but also the transferability of this concept to a more functionally complex and technologically relevant system.

At the same time, the quantitative release kinetics of gentamicin from PLA–Gen–MS require separate investigation, as the biogenic mineral phase may modify the diffusion behavior of the antibiotic. Thus, the obtained data demonstrate that PLA–Gen–MS successfully combines structural integrity, FDM suitability, local antibacterial functionality, and the potential for bioactive ionic effects within a single filament platform.

The results of the in vivo study complemented the physicochemical and microbiological data by demonstrating a favorable preliminary biocompatibility profile of PLA–Gen–MS. In the subcutaneous implantation model, the material was characterized by a more rapid reduction in inflammatory infiltration compared with the TiLOOP^®^ reference implant, the absence of a pronounced foreign body reaction, and the absence of multinucleated giant cells by Day 14 of observation. This dynamic indicates a physiological tissue response and the absence of signs suggesting a transition of inflammation to a chronic phase.

An uncontrolled inflammatory response to implantable materials may lead to fibrous encapsulation, impaired integration of the implant with surrounding tissues, and reduced regenerative potential [74,75]. In this context, the more favorable tissue response observed for PLA–Gen–MS should be interpreted as an integrated response to the composite system rather than as the effect of a single component alone. This response may be associated with the biocompatibility of the PLA matrix [76], the release of bioactive ions from the mollusk shell-derived mineral phase [65,77], and the local antimicrobial effect of gentamicin, which may reduce the risk of persistent microbial contamination and sustained inflammation.

More specifically, the ion-mediated contribution of the mineral phase may be related mainly to the release of Ca, P, Sr, and Mg ions. Ca and P are primarily relevant to mineralization-related processes, apatite formation, and osteoconductive support, whereas Sr^2+^ and Mg^2+^ may additionally participate in the regulation of the local osteoimmune microenvironment. Sr^2+^-functionalized biomaterials have been reported to promote macrophage polarization toward reparative M2-like phenotypes and to support the secretion of regenerative mediators involved in osteogenesis and angiogenesis [65,78]. Similarly, Mg^2+^ has been shown to downregulate M1-associated inflammatory responses, reduce TNF-α and IL-1β production, increase IL-10 secretion, and promote M0-to-M2 macrophage polarization, probably through reduced NF-κB activation [79].

Although detailed immunophenotyping was beyond the scope of this study, the histological findings are consistent with a favorable local tissue response. Based on published data on Sr^2+^- and Mg^2+^-mediated osteoimmunomodulation, the released mineral ions may have contributed to a microenvironment supporting the transition from early inflammation toward tissue repair; however, this assumption requires further targeted validation. Future studies comparing PLA–MS and PLA–Gen–MS under identical implantation conditions may further clarify the relative contributions of the mineral phase and gentamicin to the tissue response.

With respect to degradation, the behavior of PLA-based scaffolds is known to depend on several material- and structure-related factors, including polymer molecular weight, crystallinity, scaffold geometry, porosity, and the local physiological environment [11]. These parameters can substantially influence the rate of hydrolytic degradation and subsequent scaffold resorption. For the further development of PLA–Gen–MS for bone-regeneration applications, particular attention should therefore be given to determining whether the degradation rate of FDM-printed scaffolds is appropriately balanced with new tissue formation and progressive replacement of the scaffold during bone regeneration.

Taken together, these findings support the relevance of PLA–Gen–MS as a multifunctional filament-based system for regenerative applications, where biodegradability, bioactivity, antibacterial functionality, processability, and a controlled tissue response are key material requirements [74,75,77].

An important finding of the present work is that the functional properties of PLA–Gen–MS were confirmed in direct connection with its technological processability. Many experimental bioactive composites demonstrate promising in vitro characteristics but fail to overcome the technological barrier associated with the production of a stable filament and reproducible FDM printing of three-dimensional structures [55,67]. The selected extrusion temperature is also consistent with previously reported processing conditions for PLA-based mineral-filled filaments. For example, PLA/biphasic calcium phosphate filaments have been produced by hot-melt extrusion at 168 °C, while PLA/PCL/HAp/BT composite filaments were fabricated at 172–175 °C [80,81]. Therefore, the processing temperature used in the present study falls within the range reported for related PLA-based composite filament systems. In this study, PLA–Gen–MS was successfully produced as a continuous filament of standard diameter and used to print model objects, including lattice scaffolds. This substantially increases the applied significance of the results, as it demonstrates that the bioactivity and antibacterial functionality of the material are realized not only at the level of a laboratory composite formulation but also in the form of a technologically applicable filament platform [11,21].

Taken together, the obtained data show that the properties of PLA–Gen–MS are formed not through a simple summation of the contributions of individual components, but as a result of their functional integration within a unified compositional and technological system. The biogenic mineral phase provides a calcium-dominant multicomponent ionic profile; the PLA matrix ensures thermoplastic processability and structural integrity; gentamicin provides local antimicrobial activity after extrusion; and the resulting microstructure integrates these functions into a material suitable for FDM printing of bioactive and antibacterial scaffolds for regenerative medicine.

The present study should be considered as an initial stage of experimental validation of the target PLA–Gen–MS composition selected on the basis of the QbD rationale. At this proof-of-concept stage, the biological evaluation was focused primarily on the local tissue response to the final processed composite under implantation conditions. The subcutaneous implantation model was therefore used as a preliminary in vivo assessment and was not intended to evaluate direct bone integration, bone–material bonding, or the time required for osseointegration. This experimental strategy was also considered in light of the well-established biomedical use and biological profiles of the individual components of PLA–Gen–MS, including PLA, CaCO_3_, and gentamicin. Nevertheless, dedicated in vitro cytocompatibility testing of the final composite would provide an important complementary level of biological characterization. The extraction-based ICP-AES analysis, agar diffusion assay, and subcutaneous implantation study assessed different complementary aspects of the material under method-specific experimental conditions; accordingly, the 72 h extraction period, 24 h antibacterial endpoint, and 14-day implantation period should be interpreted within the context of their respective assays. Subsequent biological validation should include standardized cell-based assessment and orthotopic models of bone regeneration, allowing scaffold integration, bone–material interface formation, and the balance between scaffold degradation and new tissue formation to be assessed more directly.

The present 75:20:5 PLA:MS:Gen formulation represents a balanced target composition selected within the QbD framework; however, subsequent application-specific refinement may be required depending on the mechanical and biological requirements of the intended scaffold. Such refinement should preserve the balance between mechanical performance, microstructural homogeneity, mineral loading and ion-releasing functionality, antibacterial activity, and FDM processability rather than improve any single property in isolation. For example, increasing the mineral fraction may enhance the contribution of the CaCO_3_-based phase but may also promote local particle clustering, disturb continuity of the PLA matrix, and adversely affect mechanical performance and processing stability. Conversely, reducing the mineral content may improve matrix continuity and mechanical stability while decreasing the amount of the functional mineral phase and its ion-releasing potential. Therefore, any subsequent adjustment of composition or processing conditions should consider these interdependent effects simultaneously, with particular attention to maintaining uniform filler distribution together with sufficient mechanical integrity, ion-releasing and antibacterial functionality, and printability. The effects of sterilization methods on the structural, thermal, and antibacterial properties of the material should also be evaluated during further development.

Overall, the developed PLA–Gen–MS composite demonstrates that multifunctional materials for additive manufacturing can be rationally designed as integrated systems in which technological, structural, biological, and antimicrobial properties are formed in a coordinated manner. In contrast to traditional approaches focused on modifying individual characteristics, the proposed QbD strategy allows the material to be considered not merely as a modified PLA composite, but as a functional filament platform for the development of personalized bioactive and antibacterial constructs for bone regeneration.

## 5. Conclusions

In this work, a multifunctional filament platform PLA–Gen–MS was developed and experimentally validated within a Quality by Design framework for application in additive manufacturing of bioactive implantable constructs. It was demonstrated that the integration of a PLA matrix, a biogenic calcium-containing filler, and gentamicin enables the formation of a material combining technological processability, structural integrity, ion-mediated bioactive potential, and preserved antibacterial activity after melt processing.

Incorporation of the mineral phase resulted in a calcium-dominant multicomponent ionic profile, potentially relevant for the formation of a microenvironment favorable for bone regeneration, while gentamicin retained its antimicrobial activity after thermal extrusion and integration into the bulk of the polymer matrix. At the same time, the composite exhibited sufficient mechanical integrity and maintained suitability for FDM processing, despite the expected reduction in strength characteristics typical of functionalized polymer systems.

The combined results of physicochemical, technological, antibacterial, and preliminary in vivo evaluations confirm that the properties of PLA–Gen–MS arise from a coordinated compositional and technological architecture rather than a simple summation of individual component contributions. This supports the consideration of the developed material as a promising platform for the fabrication of bioactive and antibacterial scaffolds in regenerative medicine.

Overall, the proposed approach demonstrates that the application of QbD principles to the design of filament systems opens opportunities for the rational development of materials with a predefined set of properties for additive manufacturing of personalized implantable constructs.

## Figures and Tables

**Table 1:** Quality Target Product Profile (QTPP) of the multifunctional PLA-based filament.

QTPP Element	Target Value	Rationale
Intended use	Filament for additive manufacturing of biodegradable implantable constructs with antibacterial functionality	Enabling fabrication of patient-specific constructs using FDM technology
Application area	Bone regeneration with local antimicrobial support	Development of a material combining support of the regenerative microenvironment with prevention of early microbial colonization
Route of administration	Local implantation	Ensuring localized action while limiting systemic exposure
Biocompatibility	Absence of a pronounced adverse inflammatory response	Confirmation of material safety upon contact with biological tissues
Ion-releasing properties	Release of calcium-containing and associated ions from the biogenic mineral phase	Formation of a local ionic microenvironment potentially favorable for bone regeneration
Antibacterial activity	Suppression of clinically relevant bacterial pathogens	Reduction of the risk of early bacterial colonization and implant-associated infections
Mechanical integrity	Sufficient strength and stiffness for filament handling, printing, and temporary structural support	Maintaining material integrity during processing, printing, and potential implantation
Structural homogeneity	Uniform distribution of the mineral filler and gentamicin within the PLA matrix	Ensuring reproducibility of structural, mechanical, and functional properties
Geometric stability	Stable filament diameter and shape	Compatibility with FDM printing and stable material feeding
Thermal stability	Absence of significant component degradation under processing conditions	Preservation of PLA matrix structure and functional properties of active components during extrusion
Process feasibility	Production of a continuous filament and FDM fabrication of test specimens without significant technological defects	Demonstration of practical suitability of the developed material for additive manufacturing

**Table 2:** Identified Critical Quality Attributes (CQAs).

CQA	Analytical Method	Functional Significance
Filament diameter	Measurement using a micrometer/digital caliper	Ensures compatibility with fused deposition modeling (FDM) equipment and stable filament feeding
Structural and compositional homogeneity	Scanning electron microscopy (SEM)/SEM–EDS; thermogravimetric analysis (TGA); quantitative spectrophotometric determination of gentamicin sulfate	Characterizes the spatial distribution of the mineral phase and quantitatively assesses the compositional homogeneity of the mineral-associated fraction and gentamicin sulfate, supporting the consistency of structural and functional properties.
Thermal properties (Tg, Tc, Tm)	Differential scanning calorimetry (DSC)	Characterize the thermal transitions of the PLA matrix and allow assessment of the preservation of its thermal behavior after incorporation of the filler and gentamicin
Thermal stability	Thermogravimetric analysis (TGA)	Confirms the thermal resistance of composite components during extrusion and FDM processing
Mechanical integrity	Tensile testing and three-point bending	Determines the ability of the material to maintain its integrity during filament handling, printing, and use as temporary structural support
Ion release profile	Inductively coupled plasma atomic emission spectroscopy (ICP-AES)	Indicates the ability of the material to enable the formation of a calcium-dominant bioactive ionic microenvironment
Antibacterial activity	Agar diffusion method	Assesses the ability of the material to inhibit the growth of clinically relevant bacterial pathogens
Biocompatibility	In vivo subcutaneous implantation	Assesses the overall tissue response and preliminary safety of the material after implantation
FDM printability	FDM printing of test specimens and visual evaluation of print quality	Confirms the technological compatibility of the filament with the additive manufacturing process

**Table 3:** Critical Material Attributes (CMAs) and Critical Process Parameters (CPPs) Affecting CQAs.

Category	Parameter	Potential Impact on CQAs
CMA	PLA molecular weight	Influences melt viscosity, filament mechanical integrity, and extrusion stability
CMA	Mineral filler particle size	Affects filler dispersion, structural homogeneity, surface quality, mechanical properties, and the bioactive potential of the mineral phase
CMA	Mineral filler content	Determines ion release and bioactive potential and affects structural homogeneity, mechanical properties, and printability of the material
CMA	Gentamicin content	Determines antibacterial activity, distribution of the active agent within the matrix, and may affect the thermal sensitivity of the composite
CPP	Extrusion temperature	Influences melt viscosity, filament formation stability, thermal stability of components, and retention of antibacterial functionality
CPP	Extrusion rate	Affects filament geometry, diameter stability, structural homogeneity, and process reproducibility
CPP	Residence time in the heating zone	Determines the cumulative thermal exposure of PLA and gentamicin, affecting thermal stability, retention of antibacterial activity, and filament quality

**Table 4:** FDM printing parameters used for processing the PLA–Gen–MS filament.

Parameter	Value
Nozzle temperature	190 °C
Build plate temperature	50 °C
Printing speed	40 mm/s
Nozzle diameter	0.4 mm
Layer height	0.2 mm

**Table 5:** Homogeneity assessment of the dry PLA–Gen–MS blend prior to melt extrusion.

Sample	Residual Mass at 600 °C by TGA, wt.%	Gentamicin Sulfate Content, wt.%
1	20.15	5.254
2	18.71	4.694
3	22.41	4.682
4	20.55	5.470
5	20.71	4.874
6	19.25	5.112
7	18.66	4.983
Mean ± SD	20.06 ± 1.33	5.010 ± 0.291
RSD, %	6.64	5.81
Nominal content, wt.%	20.00	5.00

**Table 6:** Extrusion parameters and macroscopic characteristics of the target PLA–Gen–MS composite filament.

Parameter	Value	Technological Significance
Investigated extrusion temperature range	160–190 °C	Used as an empirical processing window to select conditions for stable filament formation
Selected extrusion temperature	172 °C	Ensures the most stable formation of a continuous filament
Screw rotation speed	2 rpm	Provides a stable processing regime
Linear filament output speed	520 mm/min (8.66 mm/s)	Contributes to reproducible filament geometry
Mean filament diameter	1.75 ± 0.05 mm	Meets the requirements for FDM printing
Macroscopic appearance	Continuous filament without pronounced defects	Confirms the technological suitability of the material

**Table 7:** Summary assessment of the compositional homogeneity of the PLA–Gen–MS filament along its length.

Analyzed Parameter	Value Range, wt.%	Mean ± SD, wt.%	RSD, %
Residual mass at 600 °C	18.87–23.89	21.72 ± 2.00	9.19
Gentamicin sulfate	4.778–5.118	4.963 ± 0.123	2.49

**Table 8:** Quantitative SEM/ImageJ parameters of mineral phase distribution in the PLA–Gen–MS filament cross-section.

Parameter	Value
Mineral area fraction in the whole cross-section, %	6.752
Number of detected mineral objects in the whole cross-section	3105
D50 particle area in the whole cross-section, µm^2^	16.731
Number of equal-area ROIs	16
ROI area CV, %	0.004
ROI mineral area fraction range, %	3.198–21.684
D50 particle area in ROIs, µm^2^	17.469 ± 3.475
D10 particle area in ROIs, µm^2^	6.717 ± 0.580

**Table 9:** Main thermal parameters calculated from the TGA curves.

Material	Tonset, °C	T5%, °C	Tmax, °C	Residual Mass, %
Mollusk shell (MS)	—	Not reached	—	97.61
Gentamicin	230.5	145.6	239.0	33.75
PLA	342.5	331.5	358.8	0.11
PLA–Gen–MS	328.9	283.3	345.3	22.08

Note. —, not applicable.

**Table 10:** Main thermal transitions identified from the DSC curves.

Material	Tg, °C	Tc, °C	Tm, °C
PLA	60	106	179
PLA–Gen–MS	60	102	177
Gentamicin	—	—	—
Mollusk shell (MS)	—	—	—

Note. —, not determined.

**Table 11:** Mechanical properties of PLA and the target PLA–Gen–MS composite.

Material	Tensile Strength, MPa	Flexural Strength, MPa
PLA	50.91 ± 3.79	113.30 ± 1.67
PLA–Gen–MS	30.06 ± 0.93	41.63 ± 6.71

**Table 12:** Concentrations of the main elements detected in the aqueous extract of PLA–Gen–MS.

Element	Concentration, mg/dm^3^
Ca	284
Sr	1.15
P	0.84
Mg	0.60
Mn	0.420
Fe	0.25
Other detected elements *	<0.1

* B, Ba, Al, Zn, Cu, Cr, Ce, Ni, V, Co, Mo, La.

**Table 13:** Total clear/contact zone diameters recorded in agar diffusion assay for PLA–Gen–MS and control samples.

Sample	*S. aureus* ATCC 6538, mm	*E. coli* ATCC 25922, mm	*P. aeruginosa* ATCC 27853, mm
PLA–Gen–MS	20.3 ± 0.9	21.0 ± 0.6	21.0 ± 1.0
Gentamicin sulfate	15.3 ± 0.9	15.7 ± 0.3	20.6 ± 1.2
PLA–MS	7.0 ± 0.0	8.0 ± 0.6	7.0 ± 0.0
PLA	8.3 ± 1.0	6.7 ± 0.8	7.0 ± 1.2

**Table 14:** Semi-quantitative and morphometric assessment of tissue response after subcutaneous implantation of TiLOOP^®^ and PLA–Gen–MS.

Group	Time Point	Neovascularization	Fibrosis	Inflammatory Response	Thickness of Inflammatory Infiltration, μm
TiLOOP^®^	Day 1	0	3	3	1054.9 (1024.7–1205.4) *
	Day 3	1	3	1	2468.3 (2150.1–2638.2) *
	Day 7	3	1	1	2274.5 (2150.4–2360.3) *
	Day 10	3	1	1	408.1 (350.4–420.7) *
	Day 14	3	1	1	357.2 (284.1–360.3) *
PLA–Gen–MS	Day 1	0	3	2	1267.9 (1123.6–1297.1) *
	Day 3	1	3	3	2067.4 (1969.7–2153.2) *
	Day 7	2	3	1	749.2 (719.8–857.6) *,†
	Day 10	2	2	1	321.4 (312.6–425.1) *
	Day 14	2	2	0	0.0 †

Note. Data for inflammatory infiltration thickness are presented as median (interquartile range). Neovascularization, fibrosis, and inflammatory response were assessed using a semi-quantitative scale from 0 to 3; scoring criteria are provided in Supplementary Table S1. * *p* < 0.05 compared with the negative control. † *p* < 0.05 compared with TiLOOP^®^ at the corresponding time point.

## Data Availability

The data presented in this study are included in the article. Further inquiries can be directed to the corresponding authors.

## Supplementary Materials

The following supporting information can be downloaded at: https://www.mdpi.com/article/10.3390/pharmaceutics18091055/s1, Table S1: Semi-quantitative Histological Scoring System; Figure S1: Preparation of PLA particulates prior to dry blending and melt extrusion.; Figure S2: Representative macrophotographs of the PLA–Gen–MS composite filament used for subsequent FDM printing. Figure S3: Representative images of agar plates illustrating antibacterial activity of PLA–Gen–MS against test strains; Video S1: FDM printing process of PLA–Gen–MS filament.
