# Supplementary material for: Bioactive PLA Filament with Antibacterial and Ion-Releasing Properties for Additive Manufacturing of Bone Scaffolds: QbD-Guided Development
Source: Pharmaceutics. 2026 Aug 25;18(9):1055. doi: 10.3390/pharmaceutics18091055 (PMC13610883; doi:10.3390/pharmaceutics18091055)
Supplement: Supplementary file 1 [file pharmaceutics-18-01055-s001.zip › Supplementary Materials Figure S1-S3.pdf]

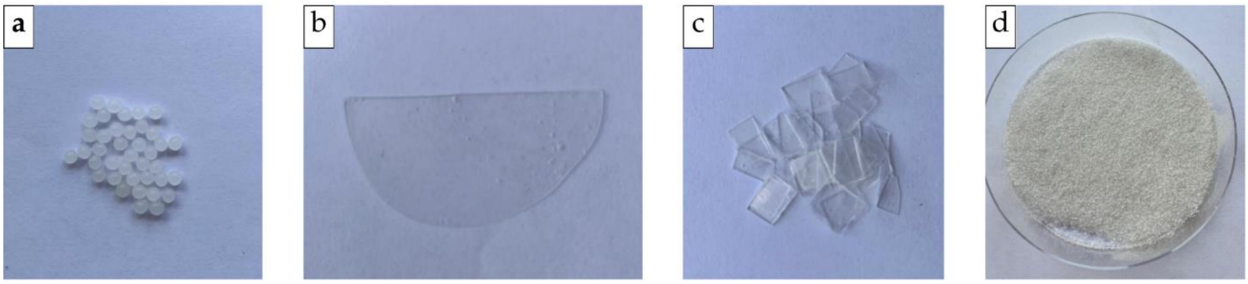

Figure S1. Preparation of PLA particulates prior to dry blending and melt extrusion. (a) Initial PLA pellets; (b) PLA plate obtained by hot pressing at 180 °C; (c) manually cut PLA fragments; (d) size-reduced and sieved PLA fraction obtained after knife milling and passage through a 500 µm sieve. The sieved PLA fraction was used for preparation of the PLA/MS/Gen dry blend.

Table S1. Semi-quantitative Histological Scoring System;

Criterion	0	1	2	3
Neovascularization	Absence of blood vessels	Vessels present only at the implant periphery	Vessels present within the implantation area, but without formation of prominent connections	Vessels present within the implantation area and forming anastomoses
Fibrosis	Absence of fibrosis	Fibrosis surrounding individual fibers/regions of the implant without bridge formation	Fibrosis surrounding individual fibers/regions of the implant with bridge formation	Scar plate covering the entire implantation area
Inflammatory response	Absence of inflammatory cells	Sparse macrophage infiltrate	Pronounced macrophage infiltrate	Macrophages and foreign body giant cells

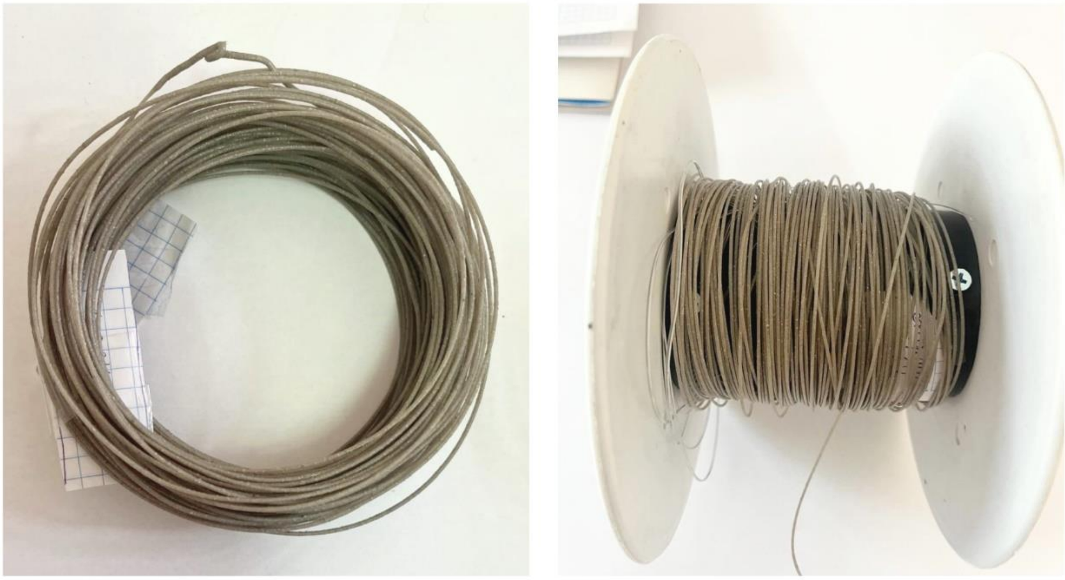

Figure S2. Representative macrophotographs of the PLA–Gen–MS composite filament used for subsequent FDM printing.

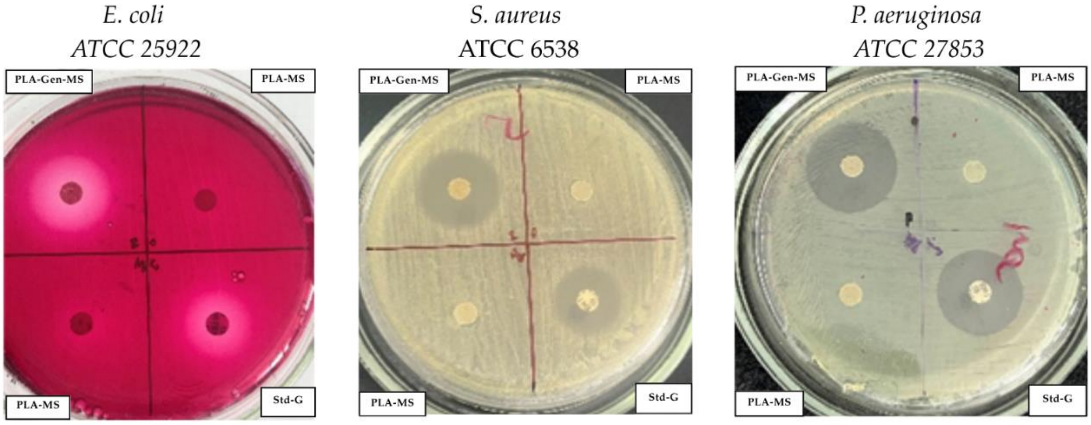

Figure S3: Representative images of agar plates illustrating antibacterial activity of PLA–Gen–MS against test strains;
